# Metabolite Profiling of Low-P Tolerant and Low-P Sensitive Maize Genotypes under Phosphorus Starvation and Restoration Conditions

**DOI:** 10.1371/journal.pone.0129520

**Published:** 2015-06-19

**Authors:** Arshid Hussain Ganie, Altaf Ahmad, Renu Pandey, Ibrahim M. Aref, Peerzada Yasir Yousuf, Sayeed Ahmad, Muhammad Iqbal

**Affiliations:** 1 Department of Botany, Faculty of Science, Hamdard University, New Delhi, 110062, India; 2 Department of Botany, Aligarh Muslim University, Aligarh, 202002, India; 3 Division of Plant Physiology, Indian Agricultural Research Institute, New Delhi, 110012, India; 4 Department of Plant Production, College of Food and Agricultural Science, P.O. Box 2460, King Saud University, Riyadh, 11451, Saudi Arabia; 5 Department of Pharmacognosy & Pharmaceutical Chemistry, Faculty of Pharmacy, Jamia Hamdard, New Delhi, 110062, India; University of Tsukuba, JAPAN

## Abstract

**Background:**

Maize (*Zea mays* L.) is one of the most widely cultivated crop plants. Unavoidable economic and environmental problems associated with the excessive use of phosphatic fertilizers demands its better management. The solution lies in improving the phosphorus (P) use efficiency to sustain productivity even at low P levels. Untargeted metabolomic profiling of contrasting genotypes provides a snap shot of whole metabolome which differs under specific conditions. This information provides an understanding of the mechanisms underlying tolerance to P stress and the approach for increasing P-use-efficiency.

**Methodology/Principal Findings:**

A comparative metabolite-profiling approach based on gas chromatography-mass spectrometry (GC/MS) was applied to investigate the effect of P starvation and its restoration in low-P sensitive (HM-4) and low-P tolerant (PEHM-2) maize genotypes. A comparison of the metabolite profiles of contrasting genotypes in response to P-deficiency revealed distinct differences among low-P sensitive and tolerant genotypes. Another set of these genotypes were grown under P-restoration condition and sampled at different time intervals (3, 5 and 10 days) to investigate if the changes in metabolite profile under P-deficiency was restored. Significant variations in the metabolite pools of these genotypes were observed under P-deficiency which were genotype specific. Out of 180 distinct analytes, 91 were identified. Phosphorus-starvation resulted in accumulation of di- and trisaccharides and metabolites of ammonium metabolism, specifically in leaves, but decreased the levels of phosphate-containing metabolites and organic acids. A sharp increase in the concentrations of glutamine, asparagine, serine and glycine was observed in both shoots and roots under low-P condition.

**Conclusion:**

The new insights generated on the maize metabolome in resposne to P-starvation and restoration would be useful towards improvement of the P-use efficiency in maize.

## Introduction

Phosphorus (P), as phosphate, is an integral component of a number of important compounds present in plant cells, such as the sugar-phosphates used in respiration and photosynthesis and the phospholipids that make up plant membranes. It is also a component of nucleotides used in plant’s energy metabolism and in the DNA and RNA molecules. Soil P deficiency is one of the most limiting factors affecting plant growth worldwide [1]. About 5.7 billion ha of soils do not contain sufficient available P for optimum crop production [2,3]. Addition of large quantities of phosphatic fertilizers to P-deficient soils is the most obvious strategy to ameliorate P deficiency. It has been forecasted by FAO that the projected increase for phosphate as fertilizer will be up to 43.8 million tonnes per annum by 2015 and 52.9 million tonnes by 2030 [4]. Demand for P in feed is also predicted to rise, driven by large increases in animal production. The non-renewable phosphate reserves in the world are likely to exhaust in the second half of this century, indicating that research aimed at developing P-efficient plants will have pivotal role in agriculture in the coming years [5].

Maize (*Zea mays* L.) is one of the most widely cultivated crop plants, for both staple food and industrial usage worldwide. Large quantities of phosphatic fertilizer are applied to maize fields in order to maximize yields. Improving the acquisition of inorganic phosphate (Pi) and utilization efficiency of crop species is important for sustainable agriculture. Thus, it is essential to understand the mechanisms by which plants react and adapt to the P-deficient growth medium. Metabolomics, i.e. monitoring a complete set of metabolites, can significantly improve the understanding of the adaptation mechanisms and provide an integrated view of the functional status of an organism. Thus, the metabolome profile can significantly contribute to the study of stress biology in plants [6]. In the present investigation, GC-MS-based metabolic profiling of leaf and root of low-P sensitive and low-P-tolerant maize was carried out under the conditions of P-sufficiency and P-deficiency and its restoration. The data generated by this approach was analyzed through advanced multivariate statistical analysis. The primary aim of this study was to dissect the mechanism of genotypic variation in maize in response to P stress condition based on changes in metabolic pathways. In this study, a list of differentially synthesised metabolites were identified which were altered between the contrasting genotypes and hence, may act act as a good road map in advancing the nutrient-uptake-efficiency of cereals, especially in maize.

## Material and Methods

### Plant Growing Conditions and Screening for P-Stress Response

Seeds of thirty-three maize (*Zea mays* L.) genotypes (S1 Table) comprising of inbreds, hybrids and composites were procured from the Directorate of Maize Research and Division of Genetics, Indian Agricultural Research Institute, New Delhi. Seeds were surface sterilized with 0.1% HgCl_2_ and kept in 0.1 mM CaCl_2_ solution in dark with constant aeration for 2 days to initiate germination. The seeds were then rolled into paper towels and kept at 30°C CaCl_2_ solution for five days. After the emergence of coleoptile, seedlings were transplanted onto thermocol sheets (2 inch thickness) fitted on plastic container (16 L capacity). Standardised Hoagland solution (half strength) was used for initial 3 days which was replaced by full-strength solution afterwards. The solution was renewed every 3 days until symptoms appeared. The composition of the nutrient (Hoagland’s) solution was already standardized for low P (2.0 μM) and sufficient P (500 μM) so that plants had no nutrient-deficiency symptoms other than those due to P, when grown in hydroponics. The composition of nutrient solution was: phosphoric acid (concentration as per treatment), CaCl_2_ (2.25 mM), MgSO_4_ (0.75 mM), NH_4_NO_3_ (4.5 mM), KCl (2.4 mM), NaCl (1 mM) H_3_BO_3_ (0.05μM), MnCl_2_ (0.01μM), ZnSO_4_ (0. 002 μM), CuSO_4_ (0.0015 μM) NH_4_Mo_7_O_24_ (0.000075 μM) and Fe-EDTA (0.074 μM). The P treatments included sufficient (500 μM) and low (2.0 μM) P concentration. The pH of nutrient solution was maintained at 5.6 and the solution was continuously aerated using aquarium pumps throughout the experiment. The typical symptom of P deficiency was visible at 15 days after transplanting (S1 Fig). The plants were raised in glasshouse at National Phytotron Facility, New Delhi, with optimum temperature (30°C/20°C D/N), relative humidity 70% and light (natural) conditions.

For screening, following physiological and biochemical traits were recorded on 15 day old plants using standard procedures, *viz*. shoot and root biomass, leaf area, root-to-shoot ratio, root length, photosynthesis rate, chlorophyll content, tissue P concentration and uptake, acid phosphatase activity in root exudates and P uptake and utilization efficiency. The experiment was laid out in completely randomised design with two-factor factorial, treatments as main plot and genotypes as split-plot. Each experiment was replicated thrice. The data obtained from screening experiment were subjected to statistical analysis in order to select contrasting genotypes. Two-way analysis of variance (ANOVA) was done using the SAS programme. Statistical significance was determined at 5% probability level. Means were compared by the critical difference (CD at *P* = 0.05) following a significant *F—*test [7]. The genotypes were classified for P starvation tolerance by performing principal component analysis (PCA) and cluster analysis by considering the trait variability under P stress. The parameters that differentiated maize genotypes for P starvation tolerance were identified by Eigenvectors generated by PCA. The variables and genotypes were classified based on the factor loading values of genotypes and variables in PC1 and PC2.

### Plant Sampling for P-Starvation and Restoration

The genotype, PEHM-2 (V1) was identified as low-P tolerant, and HM-4 (V2) as low-P sensitive. Both the genotypes were grown for 15 days in the nutrient solution with low and sufficient P levels as mentiond above. Leaf and root samples were collected on 15^th^ day from both P treatments for metabolite profiling. One set of low-P grown plants were subjected to restoration treatment by supplying sufficient P (500 μM) on 15^th^ day. Samples of leaf and root were then collected on the 3^rd^, 6^th^ and 10^th^ day of restoration treatment. At the same time, samples from the control (sufficient P) plants were also collected. Sampling was performed in three bioreplicates from three independent experiments.

### Plant Metabolite Extraction and Derivatization

Plant metabolite extraction from leaves and roots of contrasting genotypes grown under various P treatments (low P, sufficient P, 3-day restoration, 6-day restoration and 10-day restoration) was carried out for GC-MS based metabolite profiling [8]. The second fully expanded leaf and the whole root were harvested, rinsed with distilled water, dried on blotting paper, and frozen immediately in liquid- N. The frozen samples (100 mg) were ground using mortar and pestle and extracted with HPLC-grade solvent comprising of chilled isopropanol:acetonitrile:water (3:3:2). The samples were vortexed vigorously for 10 sec followed by incubation at 70°C for 15 min. The samples were centrifuged at 12000 x g for 10 min. The supernatant was collected into a screw-top glass tube and added 1.4 mL water and 0.75 mL chloroform. The mixture was again vortexed followed by centrifugation for 10 min at 4000 x g. The methanol/water phase was dried in a SpeedVac concentrator (Heto Dry Winner DW1, 0-110-20952N, Denmark) overnight. The lower phase (chloroform/ methanol phase) containing lipophilic compounds was discarded. Carbonyl moieties were protected by methoximation using 50 μL of a 20 mg/mL solution of the corresponding methoxyamine hydrochloride in pyridine at 30°C for 90 min. Subsequently, acidic protons were derivatized with 70 μL N-methyl-N-trimethylsilyl trifluoroacetamide (MSTFA) at 37°C, respectively for 90 min.

### Analysis of Samples using GC-MS

One-microliter aliquots of these solutions were injected at a split ratio of 1:25 into a GC-MS system consisting of an AS 2000 autosampler, GC-MS system [Agilent 7890A series (Germany)] equipped with split-splitless injector and CTC-PAL auto sampler attached to an apolar HP-5MS (5% phenyl polymethylsiloxane) capillary column (30 m x 0.25 mm i.d. and 0.25 μm film thickness) and fitted to a mass detector. Tuning was done according to the instruction manual, using tris(perfluorobutyl)amine (CF43) as reference gas. Special attention was paid to the high mass resolution, which was manually improved to gain resolution up to Rf_whm_ = 2800 at m/z 614. Mass spectra were recorded from m/z 50 to 600 at 0.5 s scan^-1^ for trimethylsilylated samples (TMS). Accurate mass measurements were made using a Finnigan MAT magnetic sector field instrument (Finnigan, Bremen, Germany). Chromatography was performed using a 30 m × 250 μm DB 5-MS column (J&W Scientific, Folsom, CA). Injection temperature was 230°C, detector temperature 300°C, the interface was set to 250°C, and the ion source was adjusted to 200°C. Carrier gas (He) flow was 1 mL min^-1^. After a 5 min solvent delay time at 70°C, the oven temperature was increased at 5°C min^-1^ to 310°C, 1 min isocratic, cool-down to 70°C, followed by an additional 5 min delay. Recording of mass spectra of the next sample was obstructed due to column bleeding.

### Detection and Identification of Metabolites

Raw data (S1 File, S2 Table) obtained by GC-MS analysis was identified by comparing with the reference compounds such as Wiley library of compounds and internally compiled spectra libraries, NIST (comparison software National Institute of Standards and Technology; http://www.nist.gov/srd/mslist.htm), and the mass spectral and retention time index (RI) collection of the Golm Metabolome Database (GMD) [9,10]. Retention-time correction was done by internal reference compounds in order to minimize run-to-run errors. All chemicals were purchased from Sigma-Aldrich-Fluka (SAF, Deisenhofen, Germany) like retention time standard mixture [10 ml of 0.029% (v/v) n-dodecane, n-pentadecane, n-nonadecane, n-docosane, n-octacosane, n-dotracontane, n-hexatriacontane dissolved in pyridine], was added prior to trimethylsilylation. Mass spectral matching was manually supervised, and matches were accepted with thresholds of match 650 (with the maximum match equal to 1,000) and RI deviation; 1.0% information on the polar metabolites, using the corresponding mass spectral identifiers, can be found at http://csbdb.mpimp-golm.mpg.de/csbdb/gmd/msri/gmd_smq.html. The response ratio low-P/sufficient-P for each metabolite/MST was calculated by dividing the average metabolite concentration from low-P grown plants by the average metabolite concentration from sufficient-P grown plants.

### PCA and Statistical Analysis for Metabolite Profiling

Analysis of GC-MS data was carried out using MetaboAnalyst (www.metaboanalyst.ca) web [11]. In this software, triplicate areas of each identified metabolite in the form of comma separated excel file (.CSV format) were uploaded. After submitting the file data, integrity check of the uploaded file was done automatically by the software. MetaboAnalyst replaces zero values with a small positive value (the half of the minimum positive number detected in the data). Data filtering was carried out to prevent the possibility of errors that may occur due to mathematical transformations. After filtering, the data was normalized by pooling controlled groups. The internal data structure was transformed to a table with each row representing a tissue sample (root, leaf) from low-P sensitive and low-P tolerant genotypes and each column representing a feature (a metabolite). With the data structured in this format, we performed a row-wise data normalization. These are often applied sequentially to reduce systematic variance and improve the performance for downstream statistical analysis. Row-wise normalization aims to normalize each sample (row) so that they are comparable to each other. For row-wise normalization MetaboAnalyst supports normalization to a constant sum, normalization to a reference sample (probabilistic quotient normalization), normalization to a reference feature (an internal standard) and sample specific normalization (dry weight or tissue volume). In contrast to row-wise normalization, column-wise normalization was carried out to make each feature (column) more comparable in magnitude to each other. Creating a pooled average sample from each group normalized the binned spectra data of both low-P tolerant and low-P sensitive plants. In this investigation, we chose “normalization by creating a pooled average sample from group” for row-wise normalization and “Log normalization, autoscaling” for column-wise normalization. We used a dissimilarity measure as Spearman's rank correlation and a clustering method adopted was complete linkage. The results were presented in the form of heat map with dendrogram.

## Results

### Identification of Contrasting Genotypes by PCA and Cluster Analysis

**Fig 1:**
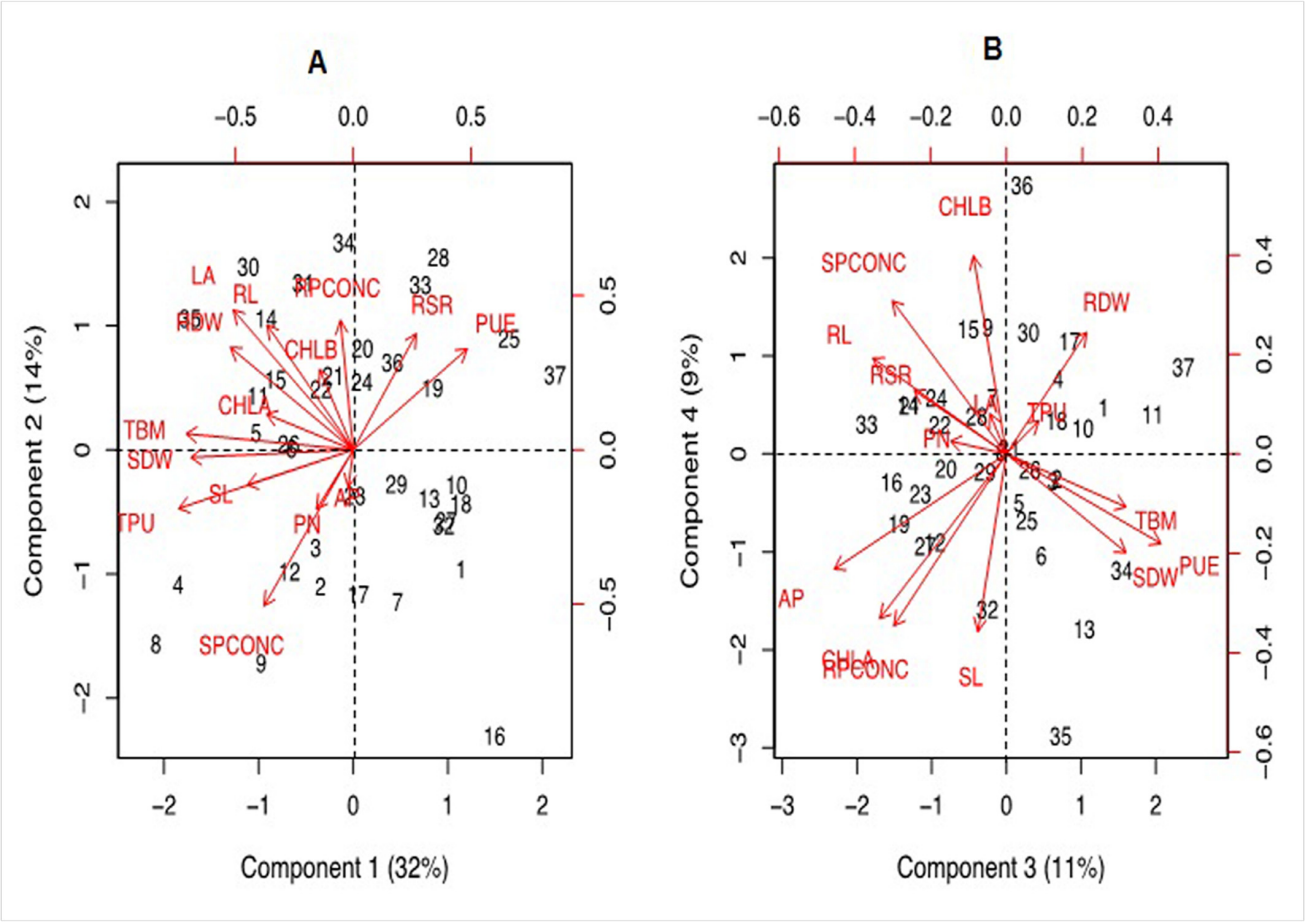
Principal component scores (PC1, PC2, PC3 and PC4) for identification of traits governing P starvation tolerance in maize genotypes grown under low (2 μM) P condition. The factor loading values for variables are indicated by red arrows radiating from the centre showing the direction (angle) and magnitude (length). Numbers indicate classification of 37 genotypes based on the factor scores of PC1 and PC2 (A) and PC3 and PC4 (B) principal components (S1 Table). Legend for variables: SL, shoot length; RL, root length; SDW, shoot dry weight; RDW, root dry weight; TBM, total biomass; RSR, root-to-shoot ratio; LA, leaf area; CHL A, chlorophyll a; CHL B, chlorophyll b; PN, photosynthesis; SPCONC, shoot P concentration; RPCONC, root P concentration; TPU, total P uptake; PUE, P use efficiency; AP, acid phosphatase activity.

Growth performance of 33 maize genotypes at sufficient and low-P levels was analysed by principal component and cluster analysis taking into consideration 15 trait variables as mentioned above. The principal component vectors, PC1 and PC2 accounted for 46% of the total variation under low-P (Fig 1A). Among the various traits in PC1, the maximum variation was explained by total P uptake (18.1%), total biomass (16.6%), shoot dry weight (15.6%), root dry weight (8.9%), leaf area (8.6%) and P use efficiency (7.7%). In PC2, maximum variability was observed for shoot P concentration (18.9%), leaf area (15.2%), root P concentration (13.1%), root length (12.2%) and root-shoot ratio (10.5%). The PC3 and PC4 also accounted for 20% of the total variation under low-P (Fig 1B). In PC3, the maximum variation was explained by acid phosphatase (18.1%), P use efficiency (14.6%) and root length (10.9%) while in PC4, chlorophyll ‘a’ and ‘b’ (18.1 and 12.6%, respectively), shoot length (14.7%7) and root and shoot P concentration (13.7% and 10.9%, respectively) governed the maximum variability. Fig 1A and 1B also shows the positive and negative scroes of all principal component vectors. Traits contributing to maximum variability were present in–PC1 and +PC2, and was classified as tolerant. PEHM-2 was identified as low-P stress tolerant alongwith its inbreds (CM 137 x CM 138) falling in the same quadrant. In +PC1 and–PC2, no trait was present and was termed as P stress sensitive. HM-4 was identified as P stress sensitive hybrid with its parents (HKI 323 x HKI 1105) +PC1 and +PC2 (Fig 1A). Similarly, the contrasting hybrids and their parents were noted for PC3 and PC4 (Fig 1B).

**Fig 2:**
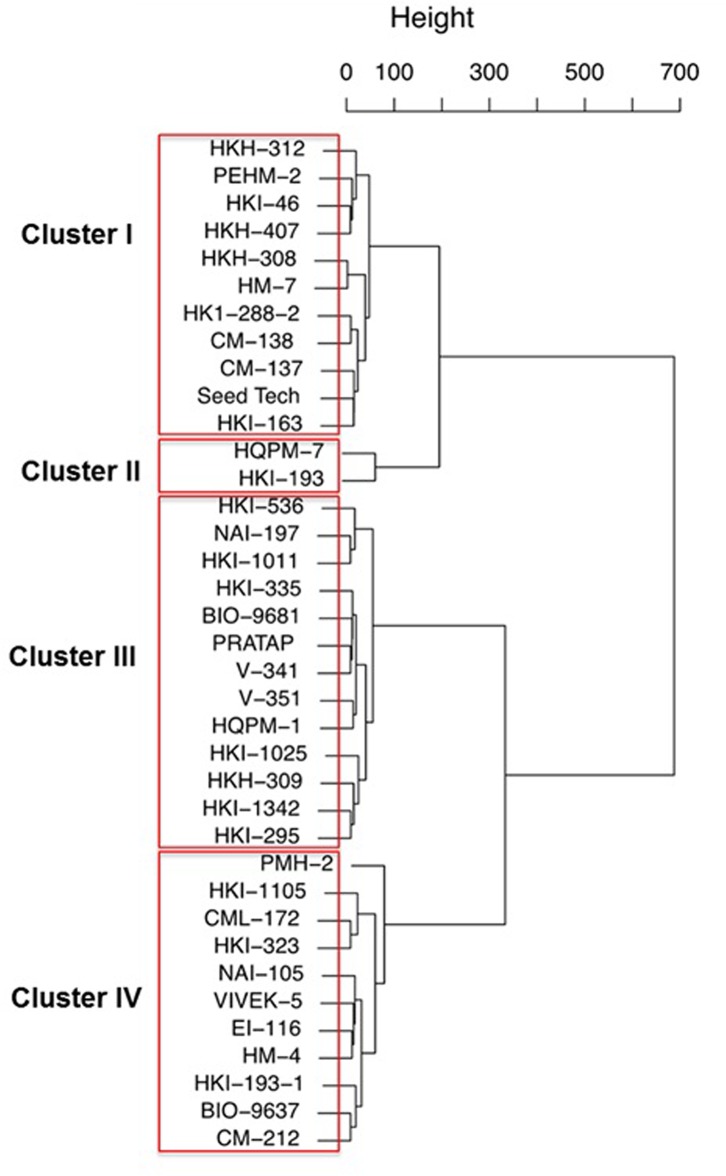
Cluster analysis of maize genotypes based on the averages of 15 trait variables grown under low (2 μM) P condition.

The genotypes were classified into four clusters using the Euclidean distances between genotypes signficantly differing in growth parameters (Fig 2). From the four clusters obtained under low-P, eleven genotypes of cluster IV were low in almost all the important traits identified by PCA. The cluster mean of eleven genotypes belonging to cluster I were highest for shoot dry weight, total plant biomass and close to the higher mean value for traits such as root length, root dry weight, leaf area, total P uptake and PUE. Two genotypes of cluster II and thirteen in cluster III had high mean values for root-shoot ratio, shoot P concentration and total P uptake.

The data of dry weight of shoot, root and total plant ranged between 0.204 g/plant (HKI-1025) to 0.712 g/plant (PEHM-2), 0.099 g/plant (HKI-1025) to 0.261 g/plant (HKH-407) g/plant and 0.303 g/plant (HKI-1025) to 0.913 g/plant (Seed Tech-2324) g/plant, respectively (Table 1). The genotypes belonging to cluster I were tolerant while those in cluster IV were sensitive to P stress. Thus, we selected PEHM-2 from cluster I and HM-4 from cluster IV for metabolomic studies under P stravation and restoration conditions.

**Table 1:** Biomass accumuation (g/plant) of 15-day-old maizegenotypes at low phosphorus (2.0 μM) and sufficient P (500 μM) treatments.

S. No.	Genotypes	P treatments		Treatment Mean
		500 μMP	2.0 μM P	
1.	EI-116	0.35	0.33	0.38
2.	NAI-197	0.46	0.44	0.48
3.	HKI-536	0.31	0.47	0.39
4.	BIO-9681	0.46	0.66	0.62
5.	HKI-288-2	0.57	0.56	0.52
6.	PRATAP	0.65	0.56	0.63
7.	VIVEK-5	0.60	0.30	0.39
8.	HKH-309	0.58	0.59	0.63
9.	HKI-335	0.33	0.45	0.41
10.	HM-4	0.45	0.31	0.43
11.	HKH-308	0.68	0.64	0.75
12.	V-341	0.22	0.45	0.37
13.	NAI-105	0.54	0.38	0.43
14.	HQPM-7	1.08	0.40	0.68
15.	PEHM-2	1.49	0.49	0.89
16.	PMH-2	0.60	0.16	0.45
17.	CML-172	0.84	0.43	0.59
18.	BIO-9637	0.54	0.30	0.40
19.	V-351	0.56	0.26	0.40
20.	HM-7	1.25	0.37	0.75
21.	HKH-312	1.56	0.38	0.71
22.	HKI-46	0.68	0.36	0.53
23.	HQPM-1	1.20	0.34	0.61
24.	HKI-1025	0.33	0.34	0.30
25.	CM-212	0.63	0.22	0.37
26.	SeedTech 2324	1.85	0.53	0.91
27.	HKI-193-1	0.84	0.19	0.42
28.	HKI-1342	0.74	0.27	0.50
29.	HKI-163	1.10	0.32	0.56
30.	HKI-193	0.44	0.50	0.41
31.	HKH-407	0.87	0.43	0.77
32.	HKI-1011	0.62	0.26	0.42
33.	HKI-295	0.44	0.27	0.34
Genotype Mean		0.72	0.39	

Values are mean of three independent replicates.

The critical difference (at P = 5%) within genotypes is 0.063, within treatments 0.022, in genotype x treatment 0.126 and the coefficient of variation (%) is 21.39.

### Principal Component Analysis (PCA) of Metabolites

**Fig 3:**
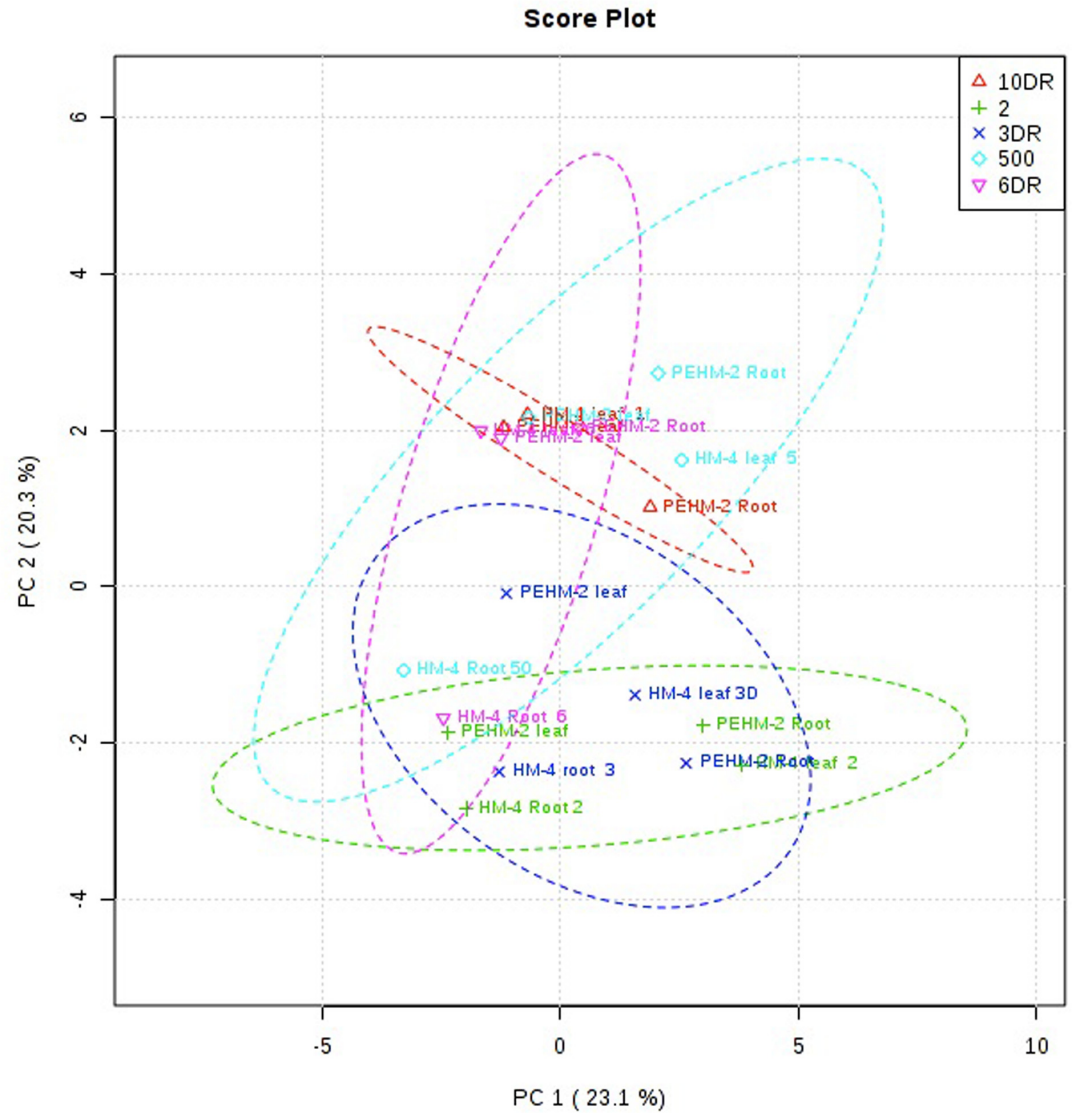
Principal component analysis (PCA) of metabolic profile of leaf and root of PEHM-2 (V1) and HM-4 (V2) maize genotypes under low P (2.0 μM) and its recovery and sufficient P (500 μM) conditions (S2 File). The PCA score plot distinguishes the metabolic profiles of low-P sensitive and low-P tolerant maize genotypes. 500 μM (open triangle), 2.0 μM (times), 3-day-restoration (open diamond, 3DR), 6-day-restoration (down triangle, 6DR) and 10-day-restoration (plus, 10DR). The first number in the data points represents treatment (500 μM P, 2 μM P, 3DR, 6DR and 10DR. Second number represents the genotype (V1 and V2). Third number indicates plant organ (leaf, S and root, R).

2-D score plot of PCA reveals a specific metabolic pattern elicited by deficiency of phosphorus in low-P tolerant (PEHM-2) and low-P sensitive (HM-4) maize genotypes. In order to reduce the large view of the dataset obtained, PCA was implemented (Fig 3, S2 File). The score plot divides the data into five ellipses on basis of degree of similarity and dissimilarity. Vigorous variations of the metabolic composition in studied tissues during phosphorus deficiency were researched. The scores of the analyses revealed a clear characteristic metabolic profile of leaf and root of PEHM-2 and HM-4 based on the positions in the 2-D plot (Fig 3). The data of the metabolic profile of the leaf and root of PEHM-2 and HM-4 under phosphorus- sufficient condition resembles the conditions of P-recovery at the 3^rd^, 6^th^ and 10^th^ day (Fig 3), because sufficient-P treatment and recoveries overlap each other, confirming that changes occurring in plants development are due to the targeted macronutrient. Low P grown HM-4 (leaf and root) falls apart. The data also revealed that the effect of low P stress is more in HM-4 than PEHM-2. It is also observed from the graph that the metabolic profile of samples taken from deficient P containing media showed more distance from each other, as compared to PEHM-2. The metabolic trend revealed by PCA was highly similar in leaf, root and restoration samples and is highly variable from treated samples.

### Metabolic Changes Under P Deficiency

**Fig 4:**
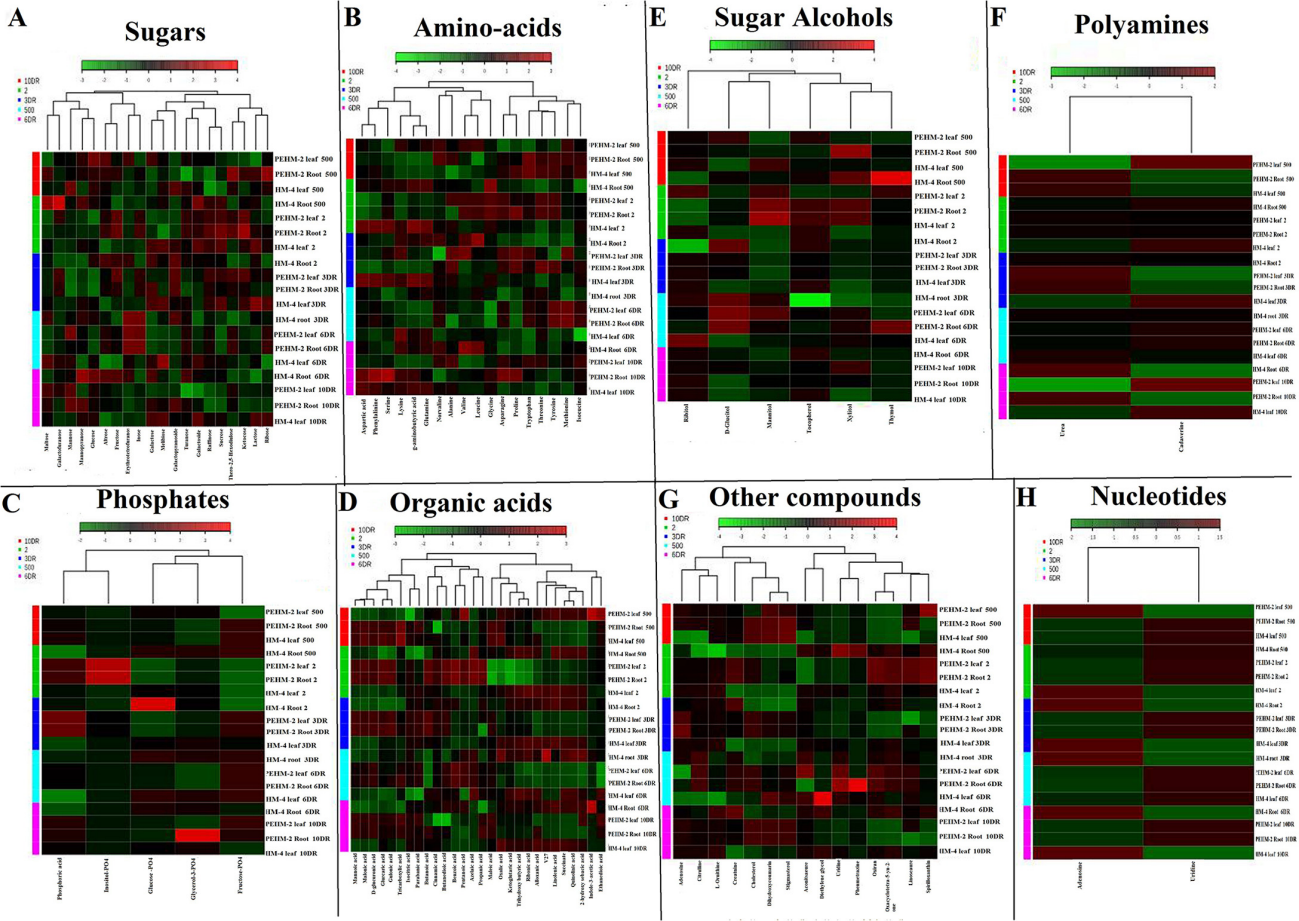
Metabolite profiles of leaf and root of PEHM-2 and HM-4 maize genotypes under sufficient P (500 μM) and low P (2.0 μM) and its restoration at 3rd, 6th, 10th day. **(S3–S10 Files).** Cluster analysis along with heat map showing changes of metabolite levels (log2) comparing leaf and root of low-P tolerant (PHEM-2) and low-P sensitive (HM-4) maize genotypes along with recovery samples. Red indicates increased metabolite levels, and green represents decreased levels (see color scale bar); Metabolites were arranged in the major groups of organic compounds: sugars, amino acids, phosphates, organic acids, sugar alcohols, polyamines, and other compounds. V1 = low-P tolerant (PEHM-2), V2 = low-P sensitive (HM-4), 500 = sufficient condition of P (500 μM), 2.0 = low P condition (2.0 μM), 3DR, 6DR, 10DR = restoration of low-P condition at 3rd, 6th and 10th day, respectively. The resulting heat map and tree figure was obtained using the Java metaboanalyst 2.0 software package. The metabolites indicated with red and green colours represent comparative metabolite concentration.

There are several groups of plant metabolites like sugars, amino acids and hydroxy acids that contain different chemical moieties, often present within the same molecule. As all these types not volatile, they have to be derivatized before GC analysis. For that purpose we used silylating reagent MSTFA (N-methyl-N-trimethylsilyltrifluoroacetamide) and the subsequent cyclization of sugars that results from derivatization is prevented by methoxyamine hydrochloride (20 mg/ml). About 180 metabolites were detected in the polar extracts of leaf and root; among them 95 known metabolites, as compared with reference libraries like NIST, Willey, were detected in the tissues of maize grown under low P (2.0 μM) and sufficient P (500 μM) conditions. Based on the reference library, the identified metabolites showed a significant response with regard to P deficiency given to low P tolerant (PEHM-2) and low P sensitive (HM-4) maize genotypes. To display the whole view of the large data sets, a heat map was generated elucidating metabolite changes under P deficiency and restoration conditions at different days. In the heat map, we compared metabolite profiles of leaf and root of both the maize genotypes grown under low P condition and P recovery with those grown under control conditions (sufficient P). Variations in metabolites under different environments are represented by fold-change values (log2), while the increased level of the metabolite under given condition is represented by red and the decreased level is indicated by green color (Fig 4, S3–S10 Files).

**Fig 5:**
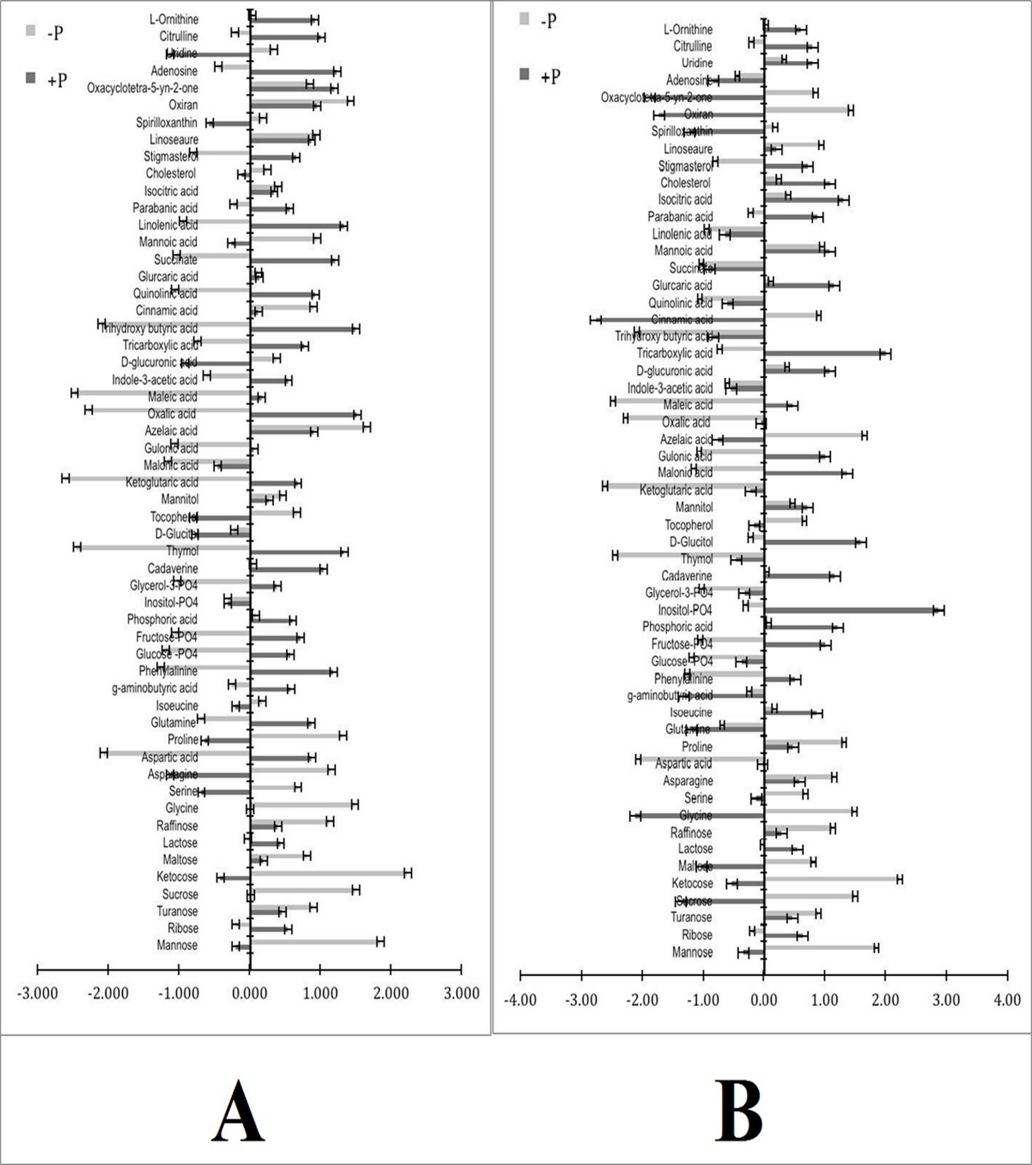
Differential metabolite profile in the leaves of PEHM-2 (V1) and HM-4 (V2) maize genotype under low (2.0 μM) and sufficient P (500 μM) conditions. The results shown are the normalized means ± SEs, and the means are the average of three independent experiments. Normalization was done with pooled control samples.

Change in the metabolite profile of leaf of PHEM-2 and HM-4 genotypes of maize, as affected by low P treatment (2.0 μM) in comparison to sufficient P condition (500 μM) is given in (Fig 5). The most commonly affected metabolites are a few di- and tri-saccharides like (raffinose, maltose, sucrose, ketocose, ribose), some amino acids (glutamine, γ-amino butyric acid, asparagine, glycine, serine, isoleucine, phenylalanine, aspartate), organic acids (ketoglutarate, parabanic acid, oxalic acid, linolenic acid, maleic acid, succinate, isocitrate, gluconic acid, cinnamic acid, glucaric acid), phosphate-containing metabolites (glucose-PO_4_, fructose-PO_4_, inositol-PO_4_, phosphoric acid), sugar alcohols (glucitol, mannitol, thymol) and some other compounds (ornithine, cholesterol, stigmasterol, oxiron, citrulline, spirollxanthin, linoseaure, adenosine). While the sucrose, raffinose, ketocose, maltose, lactose, turanose, glutamine, asparagine, glycine, serine, γ-amino butyric acid, proline, tocopherol, oxirone, spirollxanthin, linolsaeure and oxacyclotetra-5-yn-2-one were increased, ribose, mannose, aspartate, phenylalanine, isoleucine, glucose-PO_4_, fructose-PO_4_, inositol-PO_4_, phosphoric acid, gluitol, mannitol, thymol, ornithine, citrulline, chosterol, stigmosterol and adenosine decreased significantly at low-P level, compared to sufficient P level. The intensity of increase/decrease in metabolite levels differed in both the genotypes. The fold changes in the metabolite levels of leaf of both the genotypes by low P level over sufficient P level are listed in (Table 2). In low P condition, significant changes were observed in concentration of sucrose (170- fold increase), maltose (7.3- fold increase), kestose (3.7- fold increase), raffinose (3.1- fold increase) and lactose (0.5- fold) in the leaves of PHEM-2 (V1), compared with those under sufficient P condition. Contrary to this, there was a significant decrease in these metabolites in HM-4 (V2). In both the genotypes, there was a decrease in mannose and ribose (3.3 and 2.7- fold decrease, respectively in PHEM-2 and 5.35 and 0.31 in HM-4). Several amino acids, such as glycine (27-fold increase), serine (1.5-fold increase), asparagine (0.4-fold increase), proline (0.4-fold increase), glutamine (1.6-fold increase) and γ-amino butyric acid (2.9- fold increase) were significantly enhanced in PHEM-2 under low-P condition over sufficient-P condition. Contrary to these, isoleucine, aspartic acid and phenylalanine showed 15.3, 0.8, 0.4-fold decrease, respectively under low-P condition, as compared with sufficient-P condition. In HM-4, there was a significant decrease in glycine (-0.7-fold decrease), serine (5.24-fold decrease) and phenylalanine (2.42-fold decrease). However, there was an enhanced concentration of asparagine (1.94-fold increase), proline (2.74-fold increase), glutamine (0.58-fold increase) and γ-amino butyric acid (0.19-fold increase) in HM-4. While the response of phosphoric acid (3.4-fold decrease) in the leaves of PHEM-2 declined, no significant change with reference to the control appeared in HM-4. A decrease in other phosphorus-containing metabolites was also observed in both the genotypes. Organic acids exhibited large variation. In PHEM-2, a decrease was noted in the level of gulonic acid (17-fold), maleic acid (3.0-fold), glucaric acid (7.4-fold), parabanic acid (3.5-fold), isocitric acid (5.5-fold) and succinate (0.9-fold) in PHEM-2 under low-P condition, compared with the control. In HM-4 genotype, there was a slight decrease in gulonic acid (1.06-fold decrease) and parabanic acid (0.26-fold decrease). Significant increase occurred in few organic acids like oxalic acid (43.13-fold increase), ketoglutaric acid (11.55-fold increase), indole-3-accetic acid (1.11-fold increase) and isocitric acid (0.3- fold increase) in HM-4 under low-P (2.0 μM) condition, as compared with the control (500 μM), but were not affected much in PHEM-2 genotype. On the contrary, concentration of azelaic acid and cinnamic acid increased by 1.5-fold and 4.1-fold, respectively, in PEHM-2, but decreased by 2.16-fold and 0.33-fold, respectively in HM-4 under low-P condition. Significant increase was recorded in several other classes of metabolites like sugar alcohols (thymol 0.3-fold), nucleotide bases (adenosine 0.5-fold, uridine 0.7-fold), few nitrogen-containing compounds (ornithine 2.0-fold, citrulline 1.7-fold), fatty acids (stigmosterol 2.0-fold) and spirollxanthin (1.7-fold) in PEHM-2 genotype of maize under low-P condition.

**Table 2:** Fold-change in metabolite levels in the leaf of low-P tolerant (PHEM-2) and low-P sensitive (HM-4) maize genotypes under low-P and its restoration conditions, when compared with sufficient P condition.

Metabolites	PEHM-2 leaf				HM-4 leaf			
	FC	FC	FC	FC	FC	FC	FC	FC
	-P/+P	3D /+P	6D/+P	10D/+P	-P/+P	3D /+P	6D/+P	10D/+P
Mannose	-3.3^a^ **	2.21^a^ *	2.22	2.68 ^a^ *	-5.35^b^ ***	4.70^b^ ***	4.70	0.23^b^ ***
Ribose	-2.7 ^a^ **	2.37	2.61	2.37	-0.31 ^b^ ***	0.18	0.24	2.21^b^ ***
Turanose	0.7^a^ ***	0.85	0.84	0.97	1.92^b^ **	-1.73 ^b^ ***	-5.0 ^b^ ***	-5.8 ^b^ **
Sucrose	170.0 ^a^ ***	49.7 ^a^ ***	44.0 ^a^ **	39.2 ^a^ **	-1.10 ^b^ **	-0.17 ^b^ **	0.37	0.69^b^ **
Ketocose	-3.7 ^a^ **	0.07 ^a^ **	0.22 ^a^ *	0.91 ^a^ *	-4.20 ^b^ **	-1.19 ^b^ **	0.03	-0.17 ^b^ **
Maltose	7.3 ^a^ ***	1.10 ^a^ *	0.72 ^a^ **	0.87 ^a^ *	-0.79 ^b^ **	0.70	0.77	1.06^b^ **
Lactose	0.5 ^a^ **	4.21	4.66	2.86 ^a^ **	-0.05 ^b^ **	0.87^b^ **	0.88	0.93^b^ *
Raffinose	3.1 ^a^ ***	1.19 ^a^ *	1.86	0.87 ^a^ **	3.83^b^ ***	2.03	-3.3 ^b^ ***	1.15
Glycine	27	23.9 ^a^ *	18.4 ^a^ **	12. ^a^ ***	-0.70 ^b^ **	0.19	0.07^b^ ***	0.21
Serine	-1.5 ^a^ **	0.32 ^a^ *	0.97 ^a^ **	0.28 ^a^ *	-5.24 ^b^ **	2.39^b^ ***	2.75	6.94^b^ ***
Asparagine	0.4 ^a^ ***	0.57 ^a^ *	0.64 ^a^ *	0.95 ^a^ **	1.94^b^ **	1.49^b^ **	1.64	0.56^b^ **
Aspartic acid	0.8 ^a^ ***	1.41 ^a^ *	1.63 ^a^ *	1.3 ^a^ ***	107.0^b^ ***	72.61^b^ ***	16.8^b^ ***	33.1^b^ ***
Proline	0.4 ^a^ **	0.86 ^a^ *	0.51 ^a^ *	0.84 ^a^ **	2.7^b^ **	0.65^b^ **	0.72	1.06^b^ **
Glutamine	1.6 ^a^ **	0.71 ^a^ ***	0.82 ^a^ *	1.05 ^a^ *	0.58^b^ **	0.86	1.28	1.19^b^ ***
Isoeucine	15.3	-0.84	-0.71	-0.35	0.21	0.79	0.94	0.96
γ-amino-butyric acid	2.9 ^a^ **	0.86 ^a^ *	0.93	1.44 ^a^ *	0.19^b^ **	0.77^b^ *	0.74	1.34^b^ **
Phenylalinine	-0.4 ^a^ ***	0.30 ^a^ *	0.55 ^a^ *	1.03 ^a^ *	-2.4 ^b^ ***	0.63^b^ **	0.71	0.96^b^ **
Glucose-PO4	0.1 ^a^ ***	0.17 ^a^ **	0.17	1.12 ^a^ **	3.17^b^ ***	1.15	1.12	0.83^b^ **
Fructose-PO4	-1.0 ^a^ ***	0.41 ^a^ **	0.55	0.94 ^a^ *	-1.04 ^b^ ***	0.67^b^ **	0.73	1.01^b^ **
Phosphoric acid	-3.4 ^a^ **	0.03 ^a^ *	-1.35 ^a^ **	0.26	0.07^b^ ***	0.23^b^ **	0.39	1.24^b^ **
Inositol-PO4	1.7 ^a^ *	1.36 ^a^ **	0.76 ^a^ **	1.00	-0.11 ^b^ ***	0.41^b^ *	0.48	0.72^b^ **
Glycerol-3-PO4	0.1 ^a^ ***	1.03 ^a^ **	0.99	0.81	3.16^b^ ***	1.60^b^ **	2.84^b^ **	2.79^b^ **
Cadaverine	-0.2 ^a^ **	0.12 ^a^ **	0.77 ^a^ *	0.97 ^a^ **	0.03	0.37	0.74	1.43
Thymol	-0.3 ^a^ ***	0.35 ^a^ **	0.16	1.37 ^a^ **	5.33^b^ ***	1.18^b^ **	1.11	0.44^b^ ***
D-Glucitol	1.5	0.90	0.91	0.98	-0.14 ^b^ **	0.23	0.55	1.06
Tocopherol	0.1	1.20	1.13	0.95	-4.03 ^b^ ***	1.81^b^ **	1.51	1.74^b^ *
Mannitol	-3.9 ^a^ **	0.70 ^a^ **	0.85	1.14 ^a^ **	0.66	0.22	0.47	1.20
Ketoglutaric acid	0.8 ^a^ *	0.92 ^a^ *	0.97	1.02 ^a^ **	11.5^b^ ***	0.61^b^ **	0.94^b^ **	0.00
Malonic acid	2.5 ^a^ **	1.82 ^a^ *	1.45	0.91 ^a^ **	-0.84 ^b^ ***	0.76^b^ *	0.62^b^ *	1.00
Gulonic acid	-17.0 ^a^ ***	-6.08 ^a^ ***	-4.8 ^a^ ***	1.49	-1.06 ^b^ ***	1.28^b^ **	0.79^b^ **	1.10
Azelaic acid	1.5	0.72	0.87	0.99	-2.16 ^b^ **	0.69	0.94	1.07
Oxalic acid	0.2 ^a^ **	0.26 ^a^ *	0.34	0.79 ^a^ **	43.1^b^ ***	10.7^b^ ***	3.9^b^ ***	0.57^b^ ***
Maleic acid	-3.0 ^a^ **	0.30 ^a^ **	0.16	2.96 ^a^ **	-5.1^b^ **	1.04^b^ ***	1.90	2.5^b^ **
Indole-3-acetic acid	-0.5	0.69	0.83	0.95	1.1	0.55	0.75	0.85
D-glucuronic acid	1.1	0.90	0.93	1.05	0.35	0.84	0.85	0.96
Tricarboxylic acid	-1.0 ^a^ **	0.61 ^a^ **	0.94	1.01 ^a^ **	-0.37 ^b^ **	0.07^b^ ***	0.65	1.05^b^ **
Trihydroxy butyric acid	0.4	0.44	0.55	1.12	2.4^b^ **	0.24	0.22	0.36^b^ *
Cinnamic acid	4.1 ^a^ ***	1.4 ^a^ **	0.49 ^a^ **	1.09	-0.33 ^b^ **	0.93^b^ **	1.01	1.03
Quinolinic acid	1.2	0.72	0.90	1.06	1.76	1.21	1.46	1.58
Glurcaric acid	-7.4 ^a^ **	0.33	0.68	1.17	0.10^b^ *	0.83	0.57	1.2^b^ *
Succinate	-0.9 ^a^ **	0.77	0.84	0.91	1.1^b^ *	0.94	1.07^b^ *	0.98
Mannoic acid	4.3	2.67	2.16	1.34	0.87	1.09	0.93	1.08
Linolenic acid	-0.5 ^a^ **	0.68 ^a^ **	0.91	0.96	1.47	1.22	1.01	0.85
Parabanic acid	-3.5 ^a^ ***	0.72 ^a^ **	0.78	0.81	-0.26 ^b^ **	0.79	0.98	1.09^b^ **
Isocitric acid	-5.5 ^a^ **	0.81 ^a^ *	0.73	0.95 ^a^ **	0.30^b^ **	0.50	0.53	1.14^b^ **
Cholesterol	10.4 ^a^ ***	8.2^a^ **	7.85	8.08 ^a^ **	0.23^b^ *	0.54	0.90	1.05
Stigmasterol	-2.0 ^a^ **	0.93	0.95	1.15	-1.10 ^b^ **	-0.60	0.75	1.20
Linoseaure	1.4	0.55	0.84	1.08	4.4	0.88	1.06	2.7
Spirilloxanthin	-1.7 ^a^ ***	1.15 ^a^ **	0.86	0.20 ^a^ **	-0.15 ^b^ **	0.81	0.81	0.81
Oxirane	1.9	0.23	0.25	1.03	-0.83	0.65	0.89	0.88
Oxacyclotetra-5-yn-2-one	1.7	0.43	0.49	0.83	-0.45	0.53	0.90	0.90
Adenosine	-0.5 ^a^ ***	0.90	0.95	1.02 ^a^ **	0.54^b^ *	1.02 ^b^ **	0.98	0.95
Uridine	-0.7 ^a^ ***	1.08 ^a^ **	0.99	0.98 ^a^ **	0.42	0.99	1.00	1.01
Citrulline	-1.7 ^a^ **	0.46 ^a^ *	0.67 ^a^ *	1.32 ^a^ **	-0.26 ^b^ ***	0.12^b^ **	0.49	0.57^b^ **
L-Ornithine	-2.0 ^a^ **	0.81 ^a^ *	0.70	0.78 ^a^ *	0.05^b^ **	0.35^b^ **	0.45	0.95^b^ **

Values are the means of normalized data analyzed by metaboanalyst 2 software comparing the metabolite pools of low P- tolerant and low P-sensitive genotypes of maize under low-P and restoration conditions. Fold change was calculated over sufficient P condition. Comparisons are made between:

^(a)^-tolerant and control

^(b)^-intolerant and control.

All the compounds are statistically significant

*-statistically significant p<0.05

**-statistically significant p<0.01

***—statistically significant p<0.001.

FC = fold change, 3D, 6D, 10D are restorations samples harvested after 3^rd^, 6^th^ and 10^th^ day respectively.

Interestingly, however, these metabolites increased in HM-4 genotype under similar conditions. Low-P treatment caused higher increase (10.4-fold increase) in the level of cholesterol in PEHM-2 than in HM-4 (0.23-fold increase). The linoseaure level in both genotypes increased significantly under low-P condition. However, the increase was higher (4.41-fold increase) in HM-4 than in PEHM-2 (1.4-fold).

**Fig 6:**
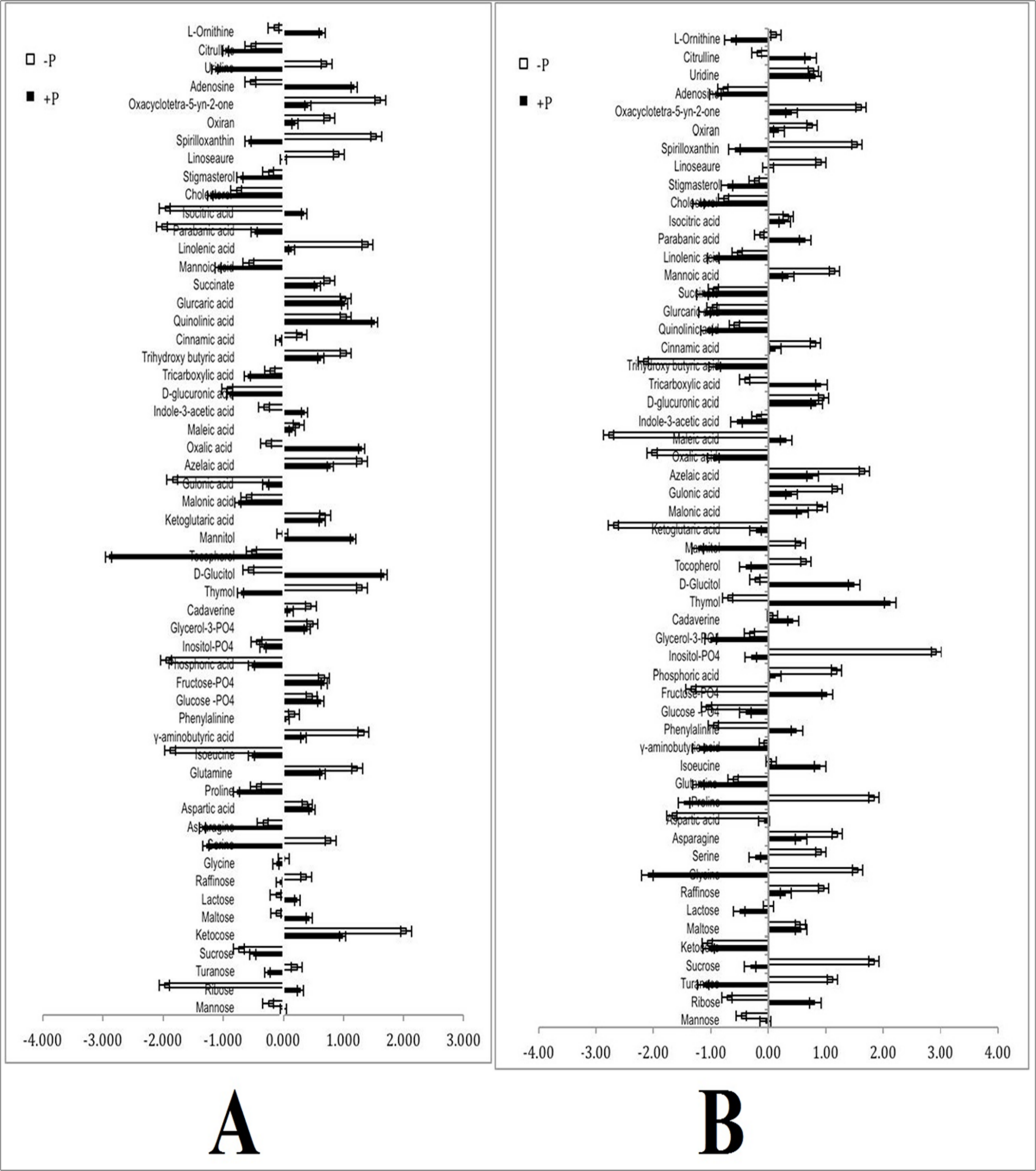
Differential metabolite profile in the root of PEHM-2 (V1) and HM-4 (V2) maize genotype under low (2.0 μM) and sufficient P (500 μM) conditions. The results shown are the normalized means ± SEs, and the means are the average of three independent experiments. Normalization was done with pooled control samples.

Similar trend of metabolite profile was observed in roots of both the genotypes under low P condition, except for a few metabolites (Fig 6). Mannose decreased by 93.28 fold in PEHM-2 root and increased by 9.87 fold in HM-4 root under low-P condition, when compared with sufficient-P condition (Table 3). Similarly, sucrose showed 1.46-fold increase in the root of PEHM-2 and 5.73-fold decrease in the roots of HM-4 under low-P condition. The change in the amino-acid profile of root is similar to that of leaf of both the genotypes. However, aspartic acid showed a 25.5-fold increase in the HM-4 root. Significant increase (3.6-fold) in phosphoric acid was observed in the roots of PEHM-2 and 9.35-fold in HM-4) whereas it was 0.07- fold in the leaf of both the genotypes under low-P condition.

**Table 3:** Fold-change in metabolite levels in the root of low-P tolerant (PHEM-2) and low-P sensitive (HM-4) maize genotypes under low-P and its restoration conditions, when compared with sufficient P condition.

Metabolite	PEHM-2 Root				HM-4 Root			
	FC	FC	FC	FC	FC	FC	FC	FC
	-P/+P	3D /+P	6D/+P	10D/+P	-P/+P	3D /+P	6D/+P	10D/+P
Mannose	-93.2^a^ ***	-361.3^a^ ***	-59.7^a^ ***	-8.9 ^a^ ***	9.8 ^a^ ***	36.1 ^a^ ***	-33.3 ^b^ **	-34.7^b^ **
Ribose	-6.8 ^a^ ***	-3.8 ^a^ **	-3.4 ^a^ **	1.41 ^a^ ***	-0.88^b^ **	-0.89 ^b^ **	-0.93 ^b^ **	0.11 ^a^ ***
Turanose	-0.87 ^a^ **	-0.85 ^a^ **	1.12	0.97	-0.99 ^b^ **	-0.03 ^b^ **	1.02 ^a^ ***	1.56
Sucrose	1.4	1.6	2.4 ^a^ **	1.53 ^a^ *	-5.7 ^b^ **	-0.8 ^b^ **	2.89	3.67
Ketocose	2.07 ^a^ **	-0.27 ^a^ ***	-0.97 ^a^ ***	-1.40 ^a^ ***	1.02 ^a^ *	0.52 ^a^ **	-0.52 ^b^ **	0.42
Maltose	-0.28 ^a^ **	0.74	2.1 ^a^ **	0.83	0.97	-1.9 ^b^ **	0.66	0.97
Lactose	-0.52 ^a^ **	2.0 ^a^ ***	-9.0 ^a^ ***	-7.2 ^a^ ***	-0.03 ^b^ **	0.91 ^a^ **	1.37	0.35 ^a^ **
Raffinose	-5.8 ^a^ **	-3.2 ^a^ **	3.76 ^a^ ***	-2.1 ^a^ **	3.1 ^a^ ***	1.48	-4.4 ^b^ ***	-7.9 ^b^ ***
Glycine	-0.09 ^a^ **	1.82 ^a^ **	2.2	2.02 ^a^ **	-0.74 ^b^ ***	0.12	-0.25 ^b^ **	0.05 ^b^ ***
Serine	-0.61 ^a^ **	0.66	1.05	1.27 ^a^ **	-3.8 ^b^ ***	-0.75 ^b^ ***	-10.8 ^b^ ***	-0.37 ^b^ ***
Asparagine	0.25 ^a^ **	0.65	0.95	1.06 ^a^ **	2.07 ^b^ **	1.76 ^b^ **	2.75	1.19 ^b^ **
Aspartic acid	0.83 ^a^ **	-0.40 ^a^ ***	-0.17 ^a^ ***	0.37	25.7 ^b^ ***	16.1 ^b^ ***	-21.2 ^b^ ***	-5.5 ^b^ ***
Proline	0.58 ^a^ **	0.93 ^a^ **	1.05	1.12	-1.2 ^b^ **	-1.09 ^b^ **	-1.07 ^b^ **	-0.81 ^b^ **
Glutamine	1.8 ^a^ *	1.33 ^a^ *	1.02	0.84 ^a^ **	0.50 ^b^ ***	0.73	0.22 ^b^ **	0.87
Isoeucine	3.5 ^a^ **	0.36 ^a^ ***	-0.20 ^a^ **	-0.14 ^a^ **	0.07	0.84	0.22	1.20
γ-aminobutyric acid	3.9 ^a^ ***	1.39 ^a^ **	0.36 ^a^ **	0.07 ^a^ ***	0.06 ^b^ **	0.60	-0.42 ^b^ ***	0.74
Phenylalinine	3.28	-13.8 ^a^ **	-16.5 ^a^ **	-9.3 ^a^ **	-1.9 ^b^ **	-2.2 ^b^ **	4.8 ^b^ ***	1.94
Glucose-PO4	0.76	5.48	0.67	0.26	2.7 ^b^ **	2.5 ^b^ **	1.40	0.58 ^b^ ***
Fructose-PO4	1	-2.5 ^a^ ***	-0.52 ^a^ **	-2.5 ^a^ ***	-1.3 ^b^ **	0.66 ^b^ *	0.66	0.78 ^b^ *
Phosphoric acid	3.7 ^a^ ***	0.25 ^a^ **	2.15	0.25 ^a^ ***	9.3 ^b^ ***	11.9 ^b^ ***	3.9 ^b^ ***	2.6^b^ ***
Inositol-PO4	1.33	1.69	0.92 ^a^ **	1.39	-9.3 ^b^ ***	-0.08 ^b^ ***	1.00	1.00
Glycerol-3-PO4	1.22	-0.04 ^a^ ***	0.69	0.40	0.32 ^b^ ***	0.45 ^b^ **	-3.5 ^b^ **	0.90 ^b^ **
Cadaverine	3.7	0.28	-13.9 ^a^ ***	13.2 ^a^ ***	0.17	-2.3	-3.3	-1.9
Thymol	-1.86 ^a^ **	-0.78 ^a^ **	-0.43 ^a^ **	0.05	-0.33	0.25	-0.33	-0.30
D-Glucitol	-0.35	0.98	-0.04 ^a^ **	0.61	-0.16	0.17	-0.70	-0.76
Tocopherol	0.18	-0.28	-0.27	-0.28	-1.6	-1.1	-0.14	0.08
Mannitol	-0.01 ^a^ ***	-0.18 ^a^ **	-0.02 ^a^ ***	0.02 ^a^ ***	-0.46 ^b^ **	0.12 ^b^ ***	0.37	-0.07 ^b^ **
Ketoglutaric acid	1.07	0.88 ^a^ **	0.92	0.77 ^a^ **	11.7 ^b^ ***	0.52 ^b^ ***	1.26	0.84 ^b^ ***
Malonic acid	0.81	1.66	1.64	1.65	1.59	1.83	1.11	1.86
Gulonic acid	6.3	5.3	2.57	4.29	2.9	3.3	1.09	1.40
Azelaic acid	1.67	-0.97	-1.07	-1.3	2.1	-0.66	-0.99	-1.1
Oxalic acid	-0.22 ^a^ **	-0.25 ^a^ **	0.44	0.32	2.10	0.51	0.56	-0.62 ^b^ **
Maleic acid	1.7 ^a^ ***	-1.87 ^a^ **	-2.1 ^a^ **	-3.6^a^ **	-8.6 ^b^ **	1.75 ^b^ ***	2.1 ^b^ **	2.42
Indole-3-acetic acid	-0.92	-1.25	8.1	8.06	0.37	0.53	0.80	0.92
D-glucuronic acid	1.05	1.14	1.22	1.25	1.15	1.30	1.10	1.11
Tricarboxylic acid	0.37 ^a^ ***	2.1 ^a^ ***	1.78 ^a^ **	2.83	-0.45 ^b^ ***	0.32 ^b^ **	0.91	1.51
Trihydroxy butyric acid	1.66	1.10	1.15	0.20	2.33	0.16	0.41	0.74
Cinnamic acid	-3.99 ^a^ **	-6.3 ^a^ **	-2.7 ^a^ **	-2.5 ^a^ **	6.3 ^b^ ***	4.06	1.39	1.49 ^b^ ***
Quinolinic acid	0.68	0.68	0.69	0.68	0.56	0.67	0.86	0.94
Glurcaric acid	1.02	0.83	0.85	0.81	0.88	0.74	0.94	0.88
Succinate	1.35	1.36	1.27	1.09	0.83 ^b^ ***	0.87	-0.65 ^a^ ***	-0.45 ^a^ ***
Mannoic acid	0.53	0.99	1.02	1.34	3.27	3.49	2.62	2.05
Linolenic acid	10.3	9.4 ^a^ **	9.52	8.9 ^a^ *	0.57	0.67	0.86	0.94
Parabanic acid	4.21	-1.04 ^a^ **	-0.58 ^a^ **	1.45	-0.24 ^b^ **	1.30	0.56	0.45
Isocitric acid	-5.6 ^a^ **	0.69	0.68	-8.4 ^a^ **	1.19	2.17	1.58	1.27
Cholesterol	0.64	0.98	0.90	0.25	0.64	0.98	0.90	0.25
Stigmasterol	0.34 ^a^ ***	1.40	1.35	-1.50 ^a^ ***	0.34	1.41	1.35	-1.51 ^b^ **
Linolseaure	328.06	-15.7	-58.5	332.4	328.06	-16.7	-62.2	353.58
Spirilloxanthin	-2.6 ^a^ **	0.01 ^a^ ***	0.56	-3.6 ^a^ **	-2.6 ^b^ ***	0.01	0.56	-3.6 ^b^ ***
Oxirane	4.01	1.13	1.54	-5.4 ^a^ **	4.01	1.13	1.54	-5.4
Oxacyclotetra-5-yn-2-one	3.92	1.55	1.20	-2.1 ^a^ **	3.92	1.55	1.20	-2.1
Adenosine	-0.4 ^a^ **	0.96 ^a^ **	1.01	1.06 ^a^ **	0.86	0.88	0.89	0.87
Uridine	-0.63 ^a^ **	0.87 ^a^ **	1.04	1.21	0.96	0.97	0.97	0.96
Citrulline	0.57	0.67	0.86	0.94	-0.27 ^b^ ***	-0.10 ^b^ ***	0.48	-2.3 ^b^ ***
L-Ornithine	-0.24 ^a^ **	1.30 ^a^ **	0.56	0.45 ^a^ **	-0.21 ^b^ ***	-0.14 ^b^ ***	-0.78 ^b^ ***	-2.0 ^b^ **

Values are the means of normalized data analyzed by metaboanalyst 2 software comparing the metabolite pools of low P- tolerant and low P-sensitive genotypes of maize under low-P and restoration conditions. Fold change was calculated over sufficient P condition. Comparisons are made between:

^a^-tolerant and control,

^b^-intolerant and control.

All the compounds are statistically significant;

*-statistically significant p<0.05,

**-statistically significant p<0.01,

***-statistically significant p<0.001.

FC = fold change, 3D, 6D, 10D are restorations samples harvested after 3^rd^, 6^th^ and 10^th^ day respectively.

Restoration of P supply to maize genotypes grown in low-P condition showed varied response on the 6th and 10^th^ day of restoration phase. In PEHM-2, resupply of P resulted in fast recovery from changes due to low-P condition. On the 6^th^ day of P-restoration phase, the metabolite profile was similar to that of plants grown under sufficient-P condition. Contrary to this, restoration of P supply to HM-4 genotype under P deficiency did not result in the recovery of metabolite profile to the level characteristic of plants grown under sufficient-P condition, even on the 10th day of restoration of P supply (Tables 2 and 3).

## Discussion

Thirty-three maize genotypes were extensively screened for P starvation tolerance using physiological and biochemical markers and two single cross hybrids were identified. From PCA, important traits contributing to P stravation tolerance were identified as reported for in other crops for abiotic tolerance [12]. Cluster analysis is a powerful tool to select an efficient genotype in a multiple-trait crop breeding [13]. From cluster analysis it was observed that the tolerant genotype, PEHM-2 belonged to cluster I which also included its parents, CM 137 and CM 138 (Fig 2). Similarly, the sensitive genotype, HM-4 and its parents were classified in cluster IV. The selected geotypes were single cross hybrids with their parents falling in the similar group justifies their performance.

**Fig 7:**
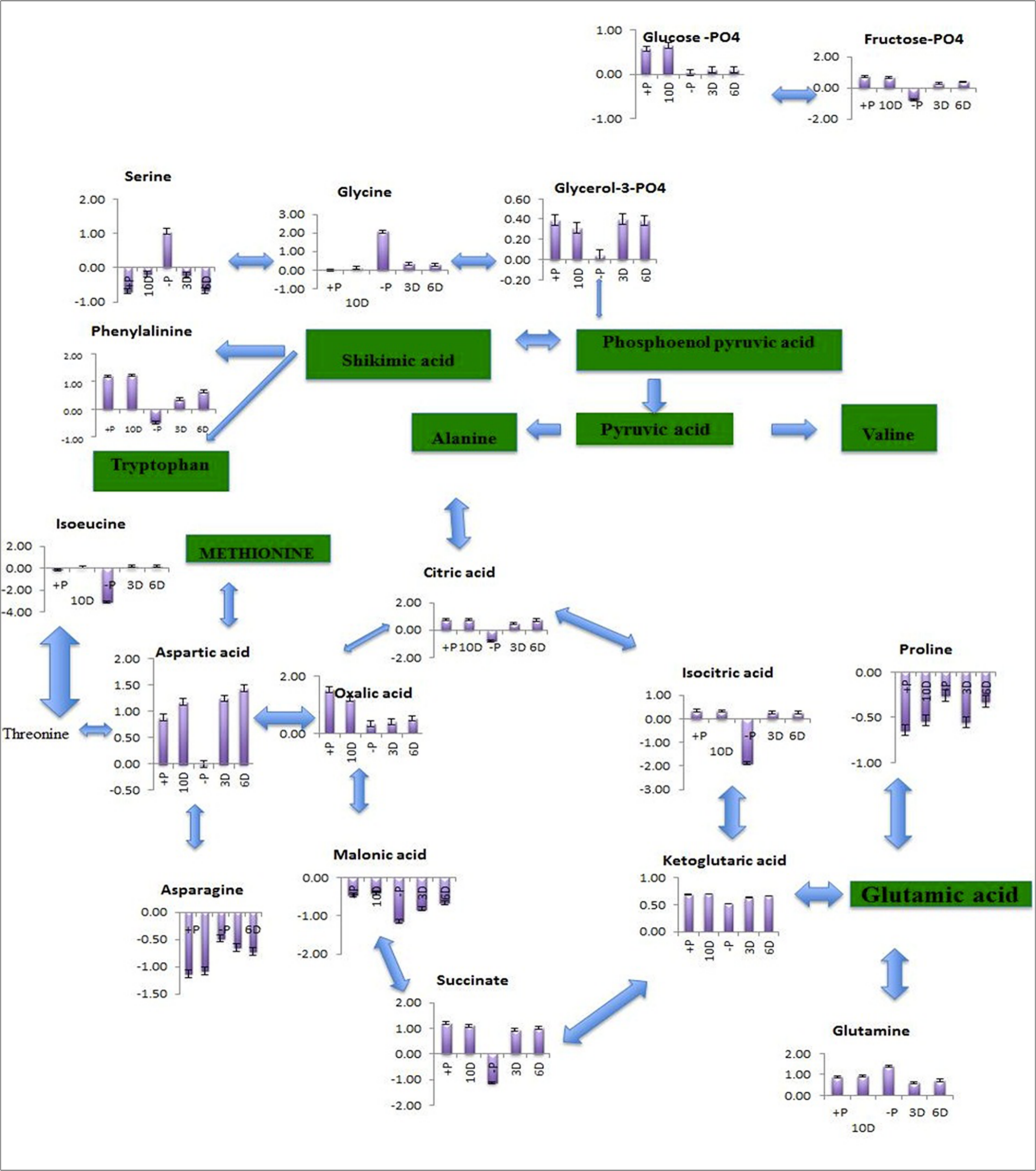
Differentially changed metabolites of carbon metabolism in leaf of maize genotypes under sufficient P (500 μM) and low P (2.0 μM) and its restoration conditions. This figure explains the effect of low P (2.0 μM) treatment and sufficient P (500 μM) on metabolic pathways such as TCA, glycolysis, amino acid metabolism. The results shown are the normalized means ± SEs, and the means are the average of three independent experiments. Normalization was done with pooled control samples. 500 = sufficient P condition, 2.0 = low P condition, 3DR, 6DR, 10DR = restorations of low P condition at 3rd, 6th, 10th day, respectively.

Comparison of the metabolite profiles of the low-P tolerant and low-P sensitive maize genotypes was carried out under P-deficient (2.0 μM P,–P) and P restoration conditions. P-sufficient (500 μM P, +P) plant population was maintained as the control. The data showed significant differences in the concentration and intensity of metabolites in response to P deficiency. The difference in metabolite concentration between the–P and +P was larger than between the +P and recovery samples. Irrespective of treatments and recovery, there was a significant difference in the metabolite profile of both the genotypes (Fig 5). However, in spite of samples taken from the same genotype, the metabolite profiles of the two different genotypes, exhibited greater differences between each other than between the different treatments and recovery samples. These results suggest that there could be more metabolic pre-adaptation in the low-P-tolerant maize genotype (PEHM-2) than in low P-sensitive genotypes (HM-4). Phosphorus deficiency enhanced the accumulation of di- and trisaccharides, particularly sucrose, maltose, raffinose, lactose, 6-kestose in the shoots of P-deficient plants. Sucrose showed the maximum (about 170-fold) increase over the control. These observations agree with some earlier reports on P-deficiency-induced increase in concentrations of maltose, raffinose, sucrose and monosaccharides (except mannose and ribose) in common beans [14], *Arabidopsis* [15,16], and *Brachiaria*, a graminaceous plant hybrid [17] and the decreased level of sucrose synthase in P-deficient maize roots [18]. The increased levels of mono-, di- and tri-saccharides imply that glycolysis may be obstructed in shoots and roots of P-deficient maize plants. It is plausible that to sustain a high level of organic-acid secretion in the proteoid roots of lupin, an increased level of glycolysis is essential under P-deficient conditions [19]. However, it is still unclear why rice did not show increased buildup of di- and polysaccharides under P-deficient conditions [17]. The possible reason for this may be that metabolic variations in glycolysis in response to P deficiency vary with species. Critical P- deficiency exhausted Pi-storage pools as signified by the severe (3.4-fold) reduction of phosphoric acid in the polar extracts of maize tissues. Under the situation of severe P deficiency, substitute resource of P for plant cells is organic P. Groups of compounds containing phosphoesters include phosphorylated metabolites (glucose-PO_4_, fructose-PO_4_), RNA, phospholipids (inositol-PO_4_). It is reported that breakdown of RNA by amplified RNase activity under Pi-limiting conditions occurs for P shortage [20]. In the severely P-deficient *Arabidopsis* plants, RNA content in shoots decreased to 13% of that in P-sufficient plants [21]. Significant quantities of organic P are contained in low- molecular- weight phosphorylated metabolites, which are roughly comparable to those in RNA or in phospholipids [22,23]. Comparative concentrations of such metabolites, like fructose-6-P, glucose-6-P, and inositol-1-P, sharply dwindled in both leaf and roots of critically P-deficient plants, and similar results were obtained for glycerol-3-P which was highly reduced in leaves and roots of severely P-deficient plants (Fig 5). Such observations, involving a sharp drop in the levels of phosphorylated metabolites, were also recorded in *Arabidopsis* and bean roots under limitation of P [24, 25]. These findings suggest that plants growing under low P-deficiency recover P from low-molecular-weight phosphorylated metabolites for vital cellular functions (Fig 7). These metabolites are key intermediates in glycolysis and involved in the synthesis of polysaccharides (fructose-6-P and glucose-6-P), phosphate-containing lipids (inositol-1-P and glycerol-3-P), nucleotides and amino acids in energy production. Therefore, exhaustion of small phosphorylated metabolites would have a critical influence not only on metabolism of carbohydrates and nitrogen but also on several other metabolic processes. The process of building up the increased storage of di- and tri-saccharides under P-limitation conditions can decrease the utilization of Pi in phosphorylating sugar containing metabolites and transform the low-molecular-weight phosphorylated metabolites to non-P-containing di- and tri-saccharides and other aromatic compounds. This might be one of the approaches to reduce Pi utilization and also have complementary benefit of acting as osmotic protectants for stressed plants. Enhanced buildup of di- and tri-saccharides for example raffinose, has been documented in plants growing under heat stress [26] and sulfate deficiency [27]. Critical temperature stress leads to increase in maltose [28] and another tri-saccharide that usually functions as building block, like 6-kestose for synthesis of fructans [29, 30]. Still, the carbohydrate that accumulates in the form of di- and tri-saccharides under P-limiting conditions cannot be consumed readily for energy metabolism without participation of Pi to offer carbon metabolites for the TCA cycle. Therefore, organic acids and amino acids will be the preferred storage form of carbon for persistence of plants growing under P-limiting conditions [17].

The reduced levels of several organic acids of TCA cycle including ketoglutarate, succinate and malic acid (Fig 7), and the reduction in the levels of aspartic acid, particularly in the tissues of low P- sensitive maize genotype (HM-4) growing under severe P- limiting condition, indicate that the non-supply of P is more apparent in the tissues of intolerant maize than in those of the P-deficient plants of tolerant PEHM-2 genotype. P deficiency has been reported to enhance the secretion of organic acids into the rhizosphere; cereals being weak secretors of organic acids in comparison to legumes under limiting P conditions [19, 31]. Plants growing under P-limiting conditions have to modify metabolic processes to consume the available carbon metabolites, like amino acids. Several amino acids will act as a complementary carbon resource for energy production, re-assimilation of the discharged ammonium and production of organic acids for secretion. Reduction in the level of several organic acids of TCA cycle was also reported in roots of P-deficient common beans [15]. Legumes are efficient secretors of organic acids [19, 31], the secretion could be a determining factor for the contribution of reduced levels of organic acids in roots. However, in *Arabidopsis* plants no reduction in the phosphorylating metabolites or in the organic acids in the TCA cycle takes place under sulfate-deficient conditions [27, 32], demonstrating that the reduction in the organic acids is characteristic to the P-deficient plant species.

Moreover, under P-limiting situations, transcript levels of genes coding for enzymes that degrade protein are up-regulated, and the genes responsible for protein synthesis are inhibited in both *Arabidopsis* and common beans [15, 33, 34]. It was found that the abundance of proteins related to protein degradation through the ubiquitin–26S proteasome pathway was enhanced in P-deficient roots of maize. Transcript level of genes involved in nitrate reduction and activities related to nitrate reduction go down in P-deficient plants [33]. Our data show that concentrations of total free amino acids in leaves and roots of severely P-deficient plants (Tables 2 and 3) are highly enhanced possibly due to increased protein degradation and repressed protein synthesis. Ammonia is produced as the byproduct of several metabolic reactions like asparagine to aspartate, glutamine to glutamate, glutamate to α-ketoglutarate and glycine to serine. In *Arabidopsis* and common beans growing under P deficiency, the transcript levels of alanine aminotransferase genes are highly enhanced [15, 33]. The levels of glutamine, glycine, serine and asparagine were highly increased in both leaves and roots of severely P-deficient plants. Other metabolites related to ammonium metabolism, like cadverine, were also elevated under low P conditions (Tables 2 and 3). The level of polyamines, like putrescine, was also enhanced in P-deficient rice cells [35]. These findings indicate that amino acids are deaminated so as to utilize them as the source of carbon metabolism, and that a simultaneous elevation in ammonium assimilation occurs in order to reduce the levels of ammonia. Activities of glutamate dehydrogenase and glutamine synthase in *Arabidopsis* plants are highly up-regulated [25], and elevated protein abundance of root glutamate dehydrogenase and glutamine synthase has been detected in maize plants growing under P-deficiency [18]. This is in line with the above observations.

Availability of carbon skeleton is a pre-requisite for the production of glutamine and asparagine. Organic acids will be highly reduced as elevated levels of glutamine and asparagine will demand more molecules α-ketoglutarate and succinate withdrawn from the TCA cycle, and hence fulfill the energy requirements of plants growing under severely P-deficient conditions. These findings showing enhanced levels of glutamine, asparagine and the metabolites participating in ammonium metabolism, as observed in our study, are also commonly detected under nitrogen and sulfur deficiency [27, 32].

Since serine and glycine are intermediates for photorespiration, the high levels of glycine and serine in the leaves and roots of plants growing under P-limiting condition show that photorespiration is enhanced [36]. Our findings also substantiate these results. Enhancements in the levels of glycine and serine were also detected in *Arabidopsis* growing under sulfur-deficiency [27], signifying that the elevation in photorespiration is a common stress response [36].

It has been observed that sugar alcohols like mannitol and glucitol increase under P-limiting condition. Mannitol plays an important role in storing the metabolites, while glucitol may act as a source of carbon skeleton for P-deficient plants [37]. Mannitol takes part in the translocation and storage of metabolites and contributes to plant resistance against salinity and osmotic pressure, while sorbitol occurs at approximately the same concentrations as sucrose in apple trees and plum trees [38]. The level of fatty acids, like cholesterol and stigmasterol, are highly reduced under P-deficient condition. Cholesterol and stigmasterol maintain membrane fluidity, but under P-limiting conditions plants scavenge P from phospholipids, disturbing the membrane fluidity and hence the formation of these phytosterols are down-regulated. Studies on soybean phosphatidylcholine bilayers indicate that all the plant sterols tested are able to regulate membrane fluidity, but with different efficiency [39].

## Conclusions

The genomic, transcriptomic, proteomic, lipidomic and ionomic studies have elucidated the mechanism of nutrient stress including that of mineral nutrients (N, P, K) in a developmental or stress-related context. Such investigations, based on analytical and bioinformatic improvements, will permit analysis of spatial and temporal nutrient- induced changes at the organ, tissue and cell levels. However, many new biomarkers or key players in nutrient signaling are expected to be identified by the ‘metabolomics’ studies. Currently, at the protein level, signaling components are analysed by interaction studies, such as the yeast two-hybrid and three-hybrid screening, BiFC, in vivo and in vitro FRET analyses. Another significant issue, i.e. to optimize sensitive methods for capturing those metabolites, which are changing in response to nutrient deficiencies, can be achieved by developing biomarkers for particular nutrient deficiency or in any other un-targeted study with the aid of metabolomics. Metabolomics also provides the snapshot of whole metabolome of an organism; hence a simultaneous determination of hundreds of metabolites is possible only with the help of these “omic” technologies. Since metabolites are solid proofs of information stored in genome of an organism, the biomarker once developed will be able to correlate it with different metabolic pathways of a plant.

## Supporting Information

S1 Fig Deficiency symptoms of low P condition in low-P tolerant (PEHM-2) and low-P sensitive maize genotypes (HM-4) along with their respective control (sufficient P). (TIF)

S1 File Mass spectrum of identified metabolites whose characteristics are defined in table provided as a supplementary file. (DOCX)

S2 File Principal component analysis (PCA) of metabolic profile of leaf and root of PEHM-2 (V1) and HM-4 (V2) maize genotypes under low P (2.0 μM), its recovery and sufficient P (500 μM) conditions. (XLS)

S3 File Sugar profile of leaf and root of PEHM-2 and HM-4 maize genotypes under sufficient P (500 μM) and low P (2.0 μM) and its restoration at 3^rd^, 6^th^, 10^th^ day. Values are normalized against the control group (+P, 500μM) (P<0.05) and log 2 transformed.
(XLS)

S4 File Amino acid profile of leaf and root of PEHM-2 and HM-4 maize genotypes under sufficient P (500 μM) and low P (2.0 μM) and its restoration at 3^rd^, 6^th^, 10^th^ day. Values are normalized against the control group (+P, 500μM) (P<0.05) and log 2 transformed.
(XLS)

S5 File Phosphate containing metabolite profile of leaf and root of PEHM-2 and HM-4 maize genotypes under sufficient P (500 μM) and low P (2.0 μM) and its restoration at 3^rd^, 6^th^, 10^th^ day. Values are normalized against the control group (+P, 500μM) (P<0.05) and log 2 transformed.
(XLS)

S6 File Organic acid profile of leaf and root of PEHM-2 and HM-4 maize genotypes under sufficient P (500 μM) and low P (2.0 μM) and its restoration at 3^rd^, 6^th^, 10^th^ day. Values are normalized against the control group (+P, 500μM) (P<0.05) and log 2 transformed.
(XLS)

S7 File Sugar alcohol profile of leaf and root of PEHM-2 and HM-4 maize genotypes under sufficient P (500 μM) and low P (2.0 μM) and its restoration at 3^rd^, 6^th^, 10^th^ day. Values are normalized against the control group (+P, 500μM) (P<0.05) and log 2 transformed.
(XLS)

S8 File Polyamine profile of leaf and root of PEHM-2 and HM-4 maize genotypes under sufficient P (500 μM) and low P (2.0 μM) and its restoration at 3^rd^, 6^th^, 10^th^ day. Values are normalized against the control group (+P, 500μM) (P<0.05) and log 2 transformed.
(XLS)

S9 File Several other metabolite that does not fall into primary group is designated as other compounds and their metabolic profile of leaf and root of PEHM-2 and HM-4 maize genotypes under sufficient P (500 μM) and low P (2.0 μM) and its restoration at 3^rd^, 6^th^, 10^th^ day. Values are normalized against the control group (+P, 500μM) (P<0.05) and log 2 transformed.
(XLS)

S10 File Nucleotide profile of leaf and root of PEHM-2 and HM-4 maize genotypes under sufficient P (500 μM) and low P (2.0 μM) and its restoration at 3^rd^, 6^th^, 10^th^ day. Values are normalized against the control group (+P, 500μM) (P<0.05) and log 2 transformed.
(XLS)

S1 Table Material used in the study to identify contrasting maize genotypes in response to P starvation tolerance. (DOC)

S2 Table Metabolites in PEHM2 and HM-4 extracts from maize plants that were significantly different under low-P and sufficient-P conditions and were tentatively identified using GC-MS. (DOCX)
