# Supplementary figures and images for: Metabolite Profiling of Low-P Tolerant and Low-P Sensitive Maize Genotypes under Phosphorus Starvation and Restoration Conditions
Source: PLoS One. 2015 Jun 19;10(6):e0129520. doi: 10.1371/journal.pone.0129520 (PMC4474700; doi:10.1371/journal.pone.0129520)

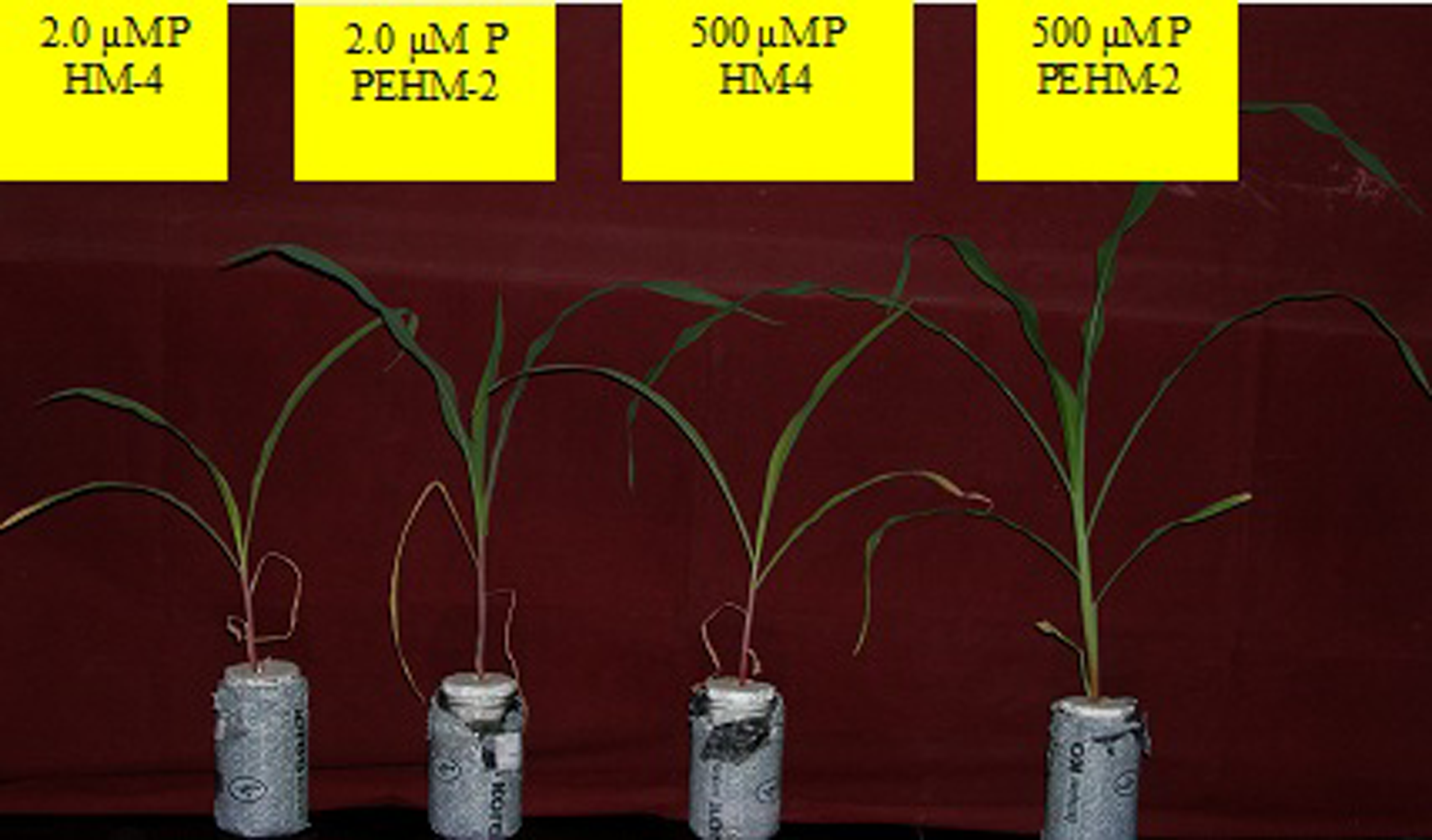

Supplement: S1 Fig — (TIF) [file pone.0129520.s001.tif]
