# Supplementary material for: Metabolite Profiling of Low-P Tolerant and Low-P Sensitive Maize Genotypes under Phosphorus Starvation and Restoration Conditions
Source: PLoS One. 2015 Jun 19;10(6):e0129520. doi: 10.1371/journal.pone.0129520 (PMC4474700; doi:10.1371/journal.pone.0129520)
Supplement: S1 File — (DOCX) [file pone.0129520.s002.docx]

Mass spectrum of identified metabolites whose characteristics are defined in table provided as a supplementary file

Spectrum 1

Spectrum 2

Spectrum 3

Spectrum 4

Spectrum 5

Spectrum 6

 Spectrum 7

Spectrum 8

Spectrum 9

Spectrum 10

Spectrum 11

Spectrum 12

 Spectrum 13

Spectrum 14

Spectrum 15

Spectrum 16

Spectrum 17

Spectrum 18

Spectrum 19

Spectrum 20

Spectrum 21

Spectrum 22

Spectrum 23

Spectrum 24

Spectrum 25

Spectrum 26

Spectrum 27

 Spectrum 28

Spectrum 29

Spectrum 30

Spectrum 31

Spectrum 32

Spectrum 33

Spectrum 34

 Spectrum 35

 Spectrum 36

 Spectrum 37

 Spectrum 38

Spectrum 39

Spectrum 40

 Spectrum 41

 Spectrum 42

Spectrum 43

Spectrum 44

Spectrum 45

Spectrum 46

Spectrum 47

Spectrum 48

Spectrum 49

Spectrum 50

Spectrum 51

Spectrum 52

Spectrum 53

Spectrum 54

Spectrum 55
