# Supplementary material for: Metabolite Profiling of Low-P Tolerant and Low-P Sensitive Maize Genotypes under Phosphorus Starvation and Restoration Conditions
Source: PLoS One. 2015 Jun 19;10(6):e0129520. doi: 10.1371/journal.pone.0129520 (PMC4474700; doi:10.1371/journal.pone.0129520)
Supplement: S1 Table — (DOC) [file pone.0129520.s012.doc]

Table S1. Material used in the study to identify contrasting maize genotypes in response to P starvation tolerance.

| **S. No.** | **Genotype** | **Pedigree/parentage** |
| --- | --- | --- |
| 1 | EI 116 | Inbred |
| 2 | NAI 197 | Inbred |
| 3 | HKI 536 | Inbred, derived from Cargill Hybrid |
| 4 | BIO 9681 | Hybrid, private |
| 5 | HKI 288-2 | Inbred |
| 6 | PRATAP | Composite, Bulk of National Pool-2 |
| 7 | VIVEK 5 | Hybrid, single cross (CM 212 x V 25) |
| 8 | HKH 309 | Hybrid |
| 9 | HKI 335 | Inbred |
| 10 | HM 4 | Hybrid, single cross (HKI 323 x HKI 1105) |
| 11 | HKH 308 | Inbred |
| 12 | V 341 | Inbred (Mexico Acc. No. 3136 F⊗-3-2-3-8-1-⊗b-#-⊗b-#-⊗b##) |
| 13 | NAI 105 | Inbred |
| 14 | HQPM 7 | Hybrid, single cross (HKI-193-1 x HKI-161) |
| 15 | PEHM 2 | Hybrid, single cross (CM 137 x CM 138) |
| 16 | PMH 2 | Hybrid, single cross (LM 15 x LM 16) |
| 17 | CML 172 | Inbred (CML: CIMMYT Maize Line) |
| 18 | BIO 9637 | Hybrid, private |
| 19 | V 351 | Inbred, V 351 (Shakti (S0) HE 25#CCB 50% F-#-⊗-1-F-⊗-4-#-B###) |
| 20 | HM 8 | Hybrid, single cross (HKI 1105 x HKI 161) |
| 21 | HKH 312 | Hybrid |
| 22 | HKI 46 | Inbred |
| 23 | HQPM 1 | Hybrid, single cross (HKI-193-1 x HKI 163) |
| 24 | HKI 1025 | Inbred |
| 25 | CM-212 | Inbred |
| 26 | Seed Tech | Hybrid, private |
| 27 | HKI 193-1 | Inbred, CML193 |
| 28 | HKI 1342 | Inbred |
| 29 | HKI 163 | Inbred, CML163 |
| 30 | HKI 193 | Inbred |
| 31 | HKH 407 | Hybrid |
| 32 | HKI 1011 | Inbred, |
| 33 | HKI 295 | Inbred, derived from composite Karnal 4 |
| 34 | CM 137 | Inbred |
| 35 | CM 138 | Inbred |
| 36 | HKI 323 | Inbred, CIMMYT Pool28 |
| 37 | HKI 1105 | Inbred, Cargil 633 |
