# Supplementary material for: Metabolite Profiling of Low-P Tolerant and Low-P Sensitive Maize Genotypes under Phosphorus Starvation and Restoration Conditions
Source: PLoS One. 2015 Jun 19;10(6):e0129520. doi: 10.1371/journal.pone.0129520 (PMC4474700; doi:10.1371/journal.pone.0129520)
Supplement: S2 Table — (DOCX) [file pone.0129520.s013.docx]

**S1 Table-1: Metabolites in PEHM2 and HM-4 extracts from maize plants that were significantly different under low-P and sufficient-P conditions and were tentatively identified using GC-MS**

| **Spectrum No.** | **t_R_ (min)** | **Metabolites** | **m/z** | **Peak no.** | **ID** | **M.F** | **Derivatized** | **Related metabolism** |
| --- | --- | --- | --- | --- | --- | --- | --- | --- |
| 1 | 27.442 | Mannose | 204 | 166 | NIST05 Lib | C_18_H_44_O_5_Si_4_ | TMS | Carbohydrate metabolism |
| 2 | 17.865 | Ribose | 217 | 81 | WILEY8 Lib | C_17_H_42_O_5_Si_4_ | TMS | Carbohydrate metabolism |
| 3 | 37.395 | Turanose | 361 | 214 | NIST05 Lib | C_33_H_78_O_11_Si_7_ | TMS | Carbohydrate metabolism |
| 4 | 35.705 | Sucrose | 362 | 167 | WILEY8 Lib | C_25_H_45_O_6_Si_8_ | TMS | Carbohydrate metabolism |
| 5 | 18.624 | Ketocose | 103 | 108 | WILEY8 Lib | C_20_H_48_N_2_O_6_Si_4_ | TMS | Carbohydrate metabolism |
| 6 | 38.715 | Maltose | 204 | 199 | NIST05 Lib | C_36_H_86_O_11_Si_8_ | TMS | Carbohydrate metabolism |
| 7 | 26.014 | Lactose | 205 | 155 | NIST05 Lib | C_36_H_86_O_11_Si_8_ | TMS | Carbohydrate metabolism |
| 8 | 16.908 | Raffinose | 171 | 95 | WILEY8 Lib | C_11_H_22_B_2_O_6_Si | TMS | Carbohydrate metabolism |
| 9 | 6.23 | Glycine | 174 | 14 | WILEY8 Lib | C_11_H_29_NOSi_3_ | TMS | Amino acid metabolism |
| 10 | 9.210 | Serine | 116 | 31 | NIST05 Lib | C_9_H_23_NO_3_Si_2_ | TMS | Amino acid metabolism |
| 11 | 15.223 | Asparagine | 188 | 72 | NIST05 Lib | C_16_H_40_NO_3_Si_2_ | (TMS)_3_ | Amino acid metabolism |
| 12 | 13.893 | Aspartic acid | 144 | 62 | NIST05 Lib | C_16_H_28_N_2_O_6_ | TMS | Amino acid metabolism |
| 13 | 14.831 | Proline | 156 | 68 | NIST05 Lib | C_11_H_23_NO_3_Si_2_ | TMS | Amino acid metabolism |
| 14 | 16.796 | Glutamine | 158 | 83 | WILEY8 Lib | C_14_H_34_N_2_O_3_Si_3_ | (TMS)_3_ | Amino acid metabolism |
| 15 | 10.041 | Isoleucine | 86 | 37 | WILEY8 Lib | C_8_H_17_NO_2_ | TMS | Amino acid metabolism |
| 16 | 17.176 | γ-amino-butyric acid | 117 | 85 | NIST05 Lib | C_10_H_25_NO_2_Si_2_ | TMS | Amino acid metabolism |
| 17 | 16.850 | Phenylalinine | 218 | 81 | NIST05 Lib | C_15_H_27_NO_2_Si_2_ | TMS | Amino acid metabolism |
| 18 | 18.625 | Glucose -PO4 | 387 | 97 | NIST05 Lib | C_25_H_64_NO_9_Psi_6_ | TMS | Glycolisis metabolism |
| 19 | 40.336 | Fructose-PO4 | 315 | 241 | NIST05 Lib | C_25_H_64_NO_9_PSi_6_ | TMS | Glycolisis metabolism |
| 20 | 19.847 | Phosphoric acid | 211 | 106 | WILEY8 Lib | C_9_H_22_NO_4_PSi_2_ | TMS | Phosphate metabolism |
| 21 | 29.712 | Inositol-PO4 | 318 | 182 | WILEY8 Lib | C_27_H_69_O_9_PSi_4_ | TMS | Phosphate metabolism |
| 22 | 28.363 | Glycerol-3-PO4 | 147 | 173 | WILEY8 Lib | C_12_H_31_NO_3_PSi_3_ | (TMS)_3_ | Glycerolipid, Carbohydrate metabolism |
| 23 | 39.483 | Cadaverine | 174 |  | NIST05 Lib | C_17_H_46_N_2_Si_4_ | TMS | Lipid metabolism |
| 24 | 22.984 | Thymol | 361 | 134 | NIST05 Lib | C_28_H_56_O_6_Si_4_ | TMS | Sugar alcohol metabolism |
| 25 | 22.300 | D-Glucitol | 103 | 128 |  | C_15_H_32_O_6_ | TMS | Sugar alcohol metabolism |
| 26 | 38.466 | Tocopherol | 502 | 224 | NIST05 Lib | C_32_H_58_O_2_Si | TMS | Sugar alcohol metabolism |
| 27 | 37.120 | Mannitol | 43 | 213 | WILEY8 Lib | C_14_H_30_O_5_ | TMS | Sugar alcohol metabolism |
| 28 | 15.983 | Ketoglutaric acid | 147 | 68 | WILEY8 Lib | C_12_H_25_NO_5_Si | (TMS)_2_ | TCA cycle metabolism |
| 29 | 20.124 | Malonic acid | 148 | 89 | NIST05 Lib | C_13_H_28_O_4_Si_2_ | TMS | Lipid metabolism |
| 30 | 28.745 | Gulonic acid | 217 | 175 | NIST05 Lib | C_18_H_42_O_6_Si_4_ | TMS | Lipid metabolism |
| 31 | 19.930 | Azelaic acid | 317 | 87 | WILEY8 Lib | C_15_H_32_O_4_Si_2_ | TMS | Lipid metabolism |
| 32 | 26.101 | Oxalic acid | 57 | 145 | NIST05 Lib | C_11_H_20_O_4_Si_2_ | TMS | TCA cycle metabolism |
| 33 | 11.299 | Maleic acid | 146 | 34 | NIST05 Lib | C_10_H_20_O_4_Si_2_ | TMS | TCA cycle metabolism |
| 34 | 23.764 | Indole-3-acetic acid | 232 | 117 | WILEY8 Lib | C_17_H_27_NO_3_Si_2_ | TMS | Lipid metabolism |
| 35 | 29.178 | D-glucuronic acid | 218 | 175 | NIST05 Lib | C_21_H_50_O_7_Si_5_ | TMS | Lipid metabolism |
| 36 | 20.593 | Tricarboxylic acid | 273 | 114 | WILEY8 Lib | C_18_H_40_O_7_Si_4_ | TMS | TCA cycle metabolism |
| 37 | 15.848 | Trihydroxy butyric acid | 292 | 76 | NIST05 Lib | C_16_H_40_O_5_Si_4_ | TMS | Lipid metabolism |
| 38 | 23.979 | Cinnamic acid | 208 | 132 | NIST05 Lib | C_11_H_12_O_4_ | TMS | Secondary metabolite metabolism |
| 39 | 38.563 | Quinolinic acid | 167 | 225 | NIST05 Lib | C_9_H_14_O_2_Si | TMS | Secondary metabolite metabolism |
| 40 | 24.356 | Glurcaric acid | 149 | 144 | WILEY8 Lib | C_24_H_58_O_8_Si_6_ | TMS | TCA cycle metabolism |
| 41 | 21.008 | Succinate | 147 |  | WILEY8 Lib | C_11_H_24_O_4_Si_2_ | TMS | TCA cycle metabolism |
| 42 | 25.610 | Mannoic acid | 204 | 142 | WILEY8 Lib | C_22_H_53_NO_7_Si_5_ | TMS | Lipid metabolism |
| 43 | 26.442 | Linolenic acid | 75 | 148 | NIST05 Lib | C_21_H_38_O_2_Si | TMS | Lipid metabolism |
| 44 | 19.718 | Parabanic acid | 100 | 116 | NIST05 Lib | C_9_H_18_N_2_O_3_Si_2_ | TMS | Lipid metabolism |
| 45 | 19.611 | Isocitric acid | 273 | 103 | NIST05 Lib | C_18_H_40_O_7_Si_4_ | (TMS)_2_ | TCA cycle metabolism |
| 46 | 41.045 | Cholesterol | 386 |  | NIST05 Lib | C_27_H_47_O | TMS | Lipid metabolism |
| 47 | 41.197 | Stigmasterol | 83 | 193 | NIST05 Lib | C_29_H_48_O | TMS | Lipid metabolism |
| 48 | 26.334 | Linoseaure | 75 | 147 | WILEY8 Lib | C_21_H_40_O_2_Si | TMS | Lipid metabolism |
| 49 | 43.581 | Spirilloxanthin | 596 | 173 | WILEY8 Lib | C_42_H_60_O_2_ | TMS | Secondary metabolite metabolism |
| 50 | 29.369 | Oxiran | 139 | 148 | WILEY8 Lib | C_11_H_23_O_2_Si_2_ | TMS | Secondary metabolite metabolism |
| 51 | 37.984 | Oxacyclotetra-5-yn-2-one | 167 | 219 | WILEY8 Lib | C_12_H_11_F_8_N_2_ | TMS | Secondary metabolite metabolism |
| 52 | 32.729 | Adenosine | 230 | 181 | NIST05 Lib | C_22_H_45_N_5_O_4_Si_4_ | (TMS)_4_ | Nucleic acid metabolism |
| 53 | 29.913 | Uridine | 217 | 201 | WILEY8 Lib | C_18_H_36_N_2_O_6_Si_3_ | (TMS)_4_ | Nucleic acid metabolism |
| 54 | 11.771 | Citrulline | 45 | 26 | WILEY8 Lib | C_18_H_45_N_3_O_3_Si_4_ | (TMS)_2_ | N_2_  metabolism |
| 55 | 20.366 | L-Ornithine | 142 | 99 | NIST05 Lib | C_17_H_44_N_2_O_2_Si_4_ | TMS | N_2_  metabolism |

t_R -_Retention time in minutes.

ID- Identification by either commercially available standard compounds in comparison with mass spectra and NIST05 and WILEY8 library

M.F- Molecular formula of the identified compounds

TMS- Derivatization was done with the help of N-Methyl-N-(trimethylsilyl)trifluoroacetamide (MSTFA)
